# Response of *Salvia officinalis* to zinc and silicon nanoparticles and pollen extract as alternates to traditional fertilizers

**DOI:** 10.3389/fpls.2024.1469691

**Published:** 2024-11-19

**Authors:** El-Sayed Mohamed El-Mahrouk, Ekramy Abdel-Moatamed Atef, Mohamed Kadry Gabr, Mahmoud Ahmed Aly, Abdallah E. Mohamed, Eman Abdelhakim Eisa, Mayank Anand Gururani

**Affiliations:** ^1^ Horticulture Department, Faculty of Agriculture, Kafrelsheikh University, Kafr El-Sheikh, Egypt; ^2^ Plant Production Department (Horticulture - Medicinal and Aromatic Plants), Faculty of Agriculture (Saba Basha), Alexandria University, Alexandria, Egypt; ^3^ Plant Production Department, Faculty of Agriculture (Saba Basha), Alexandria University, Alexandria, Egypt; ^4^ Land and Water Technologies Department, Arid Lands Cultivation Research Institute, City of Scientific Research and Technological Applications (SRTA-City), New Borg El-Arab, Alexandria, Egypt; ^5^ Department of Floriculture and Dendrology, Hungarian University of Agriculture and Life Science (MATE), Budapest, Hungary; ^6^ Botanical Gardens Research Department, Horticulture Research Institute, Agricultural Research Center (ARC), Giza, Egypt; ^7^ Biology Department, College of Science, United Arab Emirates University, Al Ain, United Arab Emirates

**Keywords:** date pollen, macroelements, sage plant, Si NPs, Zn NPs

## Abstract

*Salvia officinalis* is used in a variety of medicinal and aromatic products. The effects of various treatments on sage (*Salvia officinalis*) plants were investigated in an open-field experiment conducted between 2021 and 2022. During the experiment, ZnO nanoparticles (NPs) were used at concentrations of 1.0 and 1.5 g/L, SiO_2_ NPs were used at concentrations of 0.1 and 0.2 g/L, and date palm pollen extracts (DPE) were used at concentrations of 15 and 25 g/L, in combination with NPK fertilizers at 75%, 50%, and 25%, respectively, with a control group of 100% NPK fertilizer. A treatment consisting of 75% NPK, 15 g/L DPE, 1.0 g/L ZnO NPs, and 0.1 g/L SiO_2_ NPs significantly improved vegetative traits and essential oil yield. Compared to the control in the growing seasons of 2021 and 2022, this treatment resulted in increases in plant height, chlorophyll index, fresh and dry weights, and essential oil yield (EOY) per plant of 23.40% and 28.30%, 27.56% and 26.54%, 42.17% and 42.95%, 64.10% and 62.79%, and 93.38% and 91.08%, respectively. Combinations of 25% NPK + 25 g/L DPE + 1.5 g/L ZnO nanoparticles + 0.2 g/L SiO_2_ NPs and 75% NPK + 0.1 g/L SiO_2_ NPs produced the highest essential oil percentage (EO%). During the experimental seasons, these treatments increased EO% by 15.45% and 26.25%. In total, 58 substances were identified across the different treatments in the essential oil composition analysis. There were 11 compounds in the 25% NPK, 25 g/L DPE, 1.5 g/L ZnO NPs, and 0.2 g/L SiO_2_ NPs treatments, and 32 in the 50% NPK, 25 g/L DPE, and 0.2 g/L SiO_2_ NPs treatments. Oxygenated hydrocarbons, sesquiterpenes, and monoterpenes varied by application. Thujone, camphor, manool, and ledol were the major constituents of the EO. Leaf chemical composition, antioxidant activity, and total phenolic compounds were significantly influenced by the treatments. In combination with DPE, ZnO and SiO_2_ NPs reduced the need for higher amounts of mineral NPK fertilizers. These agents can therefore be useful for advancing sustainable agricultural practices in novel and advantageous ways.

## 1 Introduction

*Salvia officinalis* L. (commonly known as sage, kitchen sage, garden sage, culinary sage, or common sage) belongs to the family Lamiaceae. This Mediterranean plant is cultivated globally for its culinary and medicinal applications, making it one of the most prominent pharmaceutical herb (Khare et al., 2020). It is used as a native styptic, diuretic, antiseptic, tonic, anti-inflammatory, antifungal, menstruation promoter, and for spasmodic pain relief (Amer et al., 2019). The leaves are highly aromatic, and their essential oil contains more than 49 aromatic constituents, which can be useful as natural agents in cosmetics, food preservation, and pharmaceutical products (Prakash et al., 2015). Factors such as habitat, plant organ and age, genetic systems, stage of harvest (Maksimović et al., 2007), and soil conditions (Esetlili et al., 2016) strongly affect the sage essential oil percentage and chemical composition.

Nitrogen (N), phosphorus (P), and potassium (K) are essential macronutrients, frequently used in fertilization due to their critical roles in plant cell metabolism, enzymatic activity, and various physiological, chemical, and biochemical processes (Hawkesford et al., 2012). However, excessive use of chemical fertilizers can degrade soil quality, eutrophicate water bodies, and pollute air and groundwater (Congreves and Van Eerd, 2015). Furthermore, the overuse of chemical fertilizers can result in inefficient nutrient utilization and habitat disruption, posing significant challenges to sustainable agriculture. In several regions worldwide, soils have become non-responsive to NPK fertilizers, due to the decreased application of trace elements (Vanlauwe et al., 2010; Jones et al., 2013). The reduced nutrient use efficiency and environmental concerns associated with chemical fertilizer application remain significant challenges, hindering the achievement of sustainable agriculture. The use of nano-elements has been shown to enhance fertilizer efficiency (Lu et al., 2016). Zn in particular interacts with several soil elements, notably nitrogen (N) and phosphorus (P) (Loneragan and Webb, 1993). A deficiency of Zn in the soil is often linked to higher levels of available phosphorus (Takkar, 1993).

Furthermore, crops typically absorb less than half of the applied fertilizer (Chen and Wei, 2018), with the remaining amount either becoming fixed in the soil or contributing to water contamination (Liu and Lal, 2015). Specifically, N, P, and K added to the soil are lost at rates of 40-70%, 80-90%, and 50-90%, respectively (Solanki et al., 2015; Chen and Wei, 2018; Feregrino-Perez et al., 2018).

Therefore, a balanced application of macro and micronutrients is necessary. Consequently, new agricultural approaches advocate for the use of safe, ecologically acceptable products, with diverse applications. These techniques aim to enhance plant development, while mitigating environmental contamination caused by the excessive use of chemical fertilizers. The utilization of nanoparticles (NPs) derived from elements such as zinc and silicon, along with natural extracts like date palm pollen extract, represent a promising alternative to chemical fertilizers. These substitutes are both environmentally safe and friendly, offering a sustainable approach to improving agricultural productivity. Using nanotechnology to create new fertilizers is a promising approach to boost global horticultural production in a sustainable manner. This technology addresses the growing food demands of an increasing population, while promoting sustainability in the face of changing climatic conditions (Raliya et al., 2017; Feregrino-Perez et al., 2018). The application of nanoparticles (NPs) as fertilizers significantly enhances plant growth and development, while reducing the excessive use of chemical fertilizers (Acharya et al., 2020). However, numerous studies have shown that different plant species can respond variably to NPs, with effects that can be either beneficial or detrimental, depending on the nanoparticle size and dosage (El-Badri et al., 2021; Rai-Kalal and Jajoo, 2021). Nanoparticles, which range in size from 1–100 nm, offer multiple benefits, including improved plant growth, nutrition, and production (Cele, 2020). Moreover, nanotechnology has diverse applications across all stages of horticultural production, including storability, processing, packaging, and transportation. Therefore, the horticultural and food industries stand to benefit significantly from the application of nanotechnology (Swarnapriya and Vaibhao Ramesh, 2020). The interaction between nano-elements and traditional fertilizers is attributed to the high reactivity of nano-elements, which enhances the absorption of nutrients and essential materials by plants (Prasad et al., 2017). The efficiency of nanomaterials in nutrient uptake, distribution, and accumulation within plants is significantly influenced by intrinsic factors, such as particle size and surface coatings, as well as extrinsic factors, including organic matter content, soil pH, and texture. Additionally, the exposure route plays a critical role in this process (El-Ramady et al., 2018; Ma et al., 2018).

Recent reports have highlighted the effectiveness of zinc oxide nanoparticles in encouraging the development of plant species (Adrees et al., 2021; Faizan et al., 2021). Factors like pH, soil physicochemical traits, and tolerance percentage of the plant species/variety can affect the soil’s zinc availability and its impact on the crops (García-Gómez et al., 2018).Several researches indicate that foliar spray application with Zinc oxide nanoparticles (ZnONPs) is the most effective means of addressing defective microelements or the lack of them in plants (Rizwan et al., 2019; Adrees et al., 2021). During foliar spraying, plants readily and directly absorb NPs rather than depending on or utilizing the mineral fertilizers in the soil (Subramanian et al., 2015; Kah et al., 2018). Zn plays a crucial role in plants, serving as an essential component of various enzymes and acting as a key regulatory cofactor in processes such as protein synthesis, auxin production, cell division, photosynthesis, and sexual fertilization. Additionally, Zn is important for maintaining the structure and function of cell membranes (Marschner, 1995). Conversely, zinc deficiency can lead to a decline in cell growth and proliferation, negatively affecting photosynthesis, electron transport, photophosphorylation, and the leakage of electrolytes from the roots of plants (Kabata-Pendias, 2000; Auld, 2001; Genc et al., 2006). An understanding of the safety of crops and risk to the environment with application of nano zinc is still limited (Zhang et al., 2024), although, they added that there are no environmental risks from the application of ZnONPs in agriculture.

In recent times, silicon (Si) nanoparticles (NPs) have been utilized as an essential element in agricultural, mostly in arid environments, to bind other nutrients and hold onto water, thus boosting cell vigor (Sommer et al., 2006). It has been seen that utilizing Si enhances root system development, similar to the effect that a high dose of nitrogen has, resulting in improved plant chlorophyll content, photosynthesis, and quality of the product (Kah et al., 2018), thus, alleviating the adverse consequences of illnesses and abiotic pressures on plants (Farouk and Omar, 2020; Khator and Shekhawat, 2020). The electron transfer rate, stomatal conductance, and photochemical processes are all positively affected by silicon nanoparticles (SiNPs) (Ahanger et al., 2020). Silicon reduces the transpiration rate due to the thicker cuticle layer in silicon-treated plants (Rahmawati and Wulandari, 2024). Moreover, because safranal biosynthesis originates from zeaxan, the availability of Si influences the synthesis of carotenoids and the appropriate operation of chloroplasts. Silicon affects safranal biosynthesis due to its role as an intermediate in the carotenoid cycle (Pitsikas, 2016). Also, SiO_2_ NPs have distinctive characters like bioactivity, stability, and customizable porosity, therefore, SiO_2_ NPs are applied strongly in technological domains variety. However, there is no evidence to suggest that silica, the main component of Si NPs, is ecotoxic to microorganisms, fish, birds, invertebrates, and plants.

Pollen from mal tress of date palms (*Phoenix dactylifera*; Palmaceae) is widely acknowledged as being among the most effective and is utilized more often throughout the Middle East, particularly in Egypt. The various constituents present in date palm pollen, including the enzymes that are estimated by electrophoresis (Al-Helal, 1992), proteins, saponins, strolls, triterpenes, vitamins A, C, and E, macro- and micro-nutrients like N, Zn, B, Mo, Se, Fe, Mn, as well as Cu, glycosides, and carbohydrates, have several amino acids, and thirteen fatty acids (e.g., palmitic acid 34.45%), also phenylethane (8.75%), antioxidants, essential oils, flavonoids and phenols, in addition to several steroids, like, brassinosteroid (Mahran et al., 1985; Hassan, 2011; Basuny et al., 2013). Consequently, the utilization of date palm pollen significantly affects plant growth and secondary metabolism positively.

Currently, the available data on the combined effects of NPK, DPE, and NPs of ZnO and SiO2, evaluated in terms of vegetative development, EO productivity, and chemical composition, are limited. Thus, the goal of this field experiment is to assess how NPK fertilizers, ZnO and SiO2 NPs, and date palm pollen extract, affect the growth, development, essential oil productivity, and chemical and biological composition of Salvia officinalis. The study also seeks to determine a safe and affordable substitute fertilizer source that can partially substitute chemical fertilizers (NPK) to reduce pollution in the environment and guarantee the production of safe agricultural products.

## 2 Materials and methods

The study was conducted in an open field at the Experimental Farm of the Horticulture Department, Faculty of Agriculture, (Saba Basha), Alexandria University. The Experimental Farm was at Abees village, which is located in West Alexandra Governorate, Egypt, with an elevation of 2 m abovemean sea level, temperature through the experimental period of each season ranged from 25°C–37°C, and relative humidity was 55%–70%. This experiment took place during the 2021 and 2022 seasons.

### 2.1 Analysis of the soil at the experimental site

Before planting, 200 g of soil was collected at the experimental site, at various locations, from a depth of 0 to 30 cm. The samples of soil were accurately mixed into a single sample in order to evaluate their physical and chemical properties (**Table 1**).

**Table 1:** The physical and chemical parameters of the experimental site soil.

Physical parameters		Chemical parameters				Available macronutrients (mg/kg)				
Clay Sand Silt	60.33% 30.40% 09.27%	pH (1:2.5)	Organic matter %		EC (ds/m)	N		P		K
Soil texture clayey sand		8.1	2.36		1.81	799		28.3		51.6
		Anions (meq/l)				Cations (meq/l)				
		CO^–^_3_	Cl^-^	HCO^-^_3_	SO^-^_-4_	Na^+^	K^+^	Mg^++^	Ca2^++^	Zn^++^
		0.0	5.9	2.9	13.3	10.2	0.81	4.11	10.2	0.71

+, cation monovalent; ++, cation bivalent.

Following an air-drying method, the soil samples were crushed using a mortar and pestle. The samples were collected and divided into fractions of less than 2 mm, using a stainless steel test sieve (Cools and De Vos, 2011). The hydrometer (US 21 CFR 1040.10 AND 1040.11, USA) method was used to assess the distribution of the particle size (Gee and Bauder, 1986). Twenty grams of dried soil and 100 mL of deionized water were mixed in the ratio of 1:5 to obtain the soil chemical character measurements. Before the extract was filtered, the mixture was left to sit for a full day. The following measurements were made and the extract filtered: The soil EC was measured using an EC meter (MI 170, SZ egged, Hungary, Italy) (Jackson, 1973). Additionally, methods described by (Jackson, 1973) were used to estimate the concentrations of magnesium (Mg^++^), calcium (Ca^++^), and chloride (Cl^−^)_ENREF_40. The method of quantifying total carbonate and organic matter was utilized (Nelson and Sommers, 1996). The amount of accessible N was estimated by the micro Kjeldahl (DNB.1500 NP S.N. 33848 made in spain RAYPA) method (Bremner and Mulvaney, 1983). The method of Olsen and Sommers (1982) was used to estimate the available P. Zn^++^ availability was estimated using an atomic absorption spectrophotometer (AAS) (Page et al., 1982); even as Na^+^ and K^+^ were measured using a PSC 7 flame photometer (JENEWY, Staffordshire, UK) (Black et al., 1965). After 30 minutes, a pH-meter (JENEWAY3510, Staffordeshize, UK) was used to estimate the soil pH in the suspension (1:2.5, soil: distilled water) (Black et al., 1965).

### 2.2 Sowing of seeds

Sage seeds were obtained from Egypt’s Department of Medicinal and Aromatic Plants, Horticulture Research Institute, Agricultural Research Center, Ministry of Agriculture. After soaking the seeds ina 5% commercial Na hypochloride solution for five minutes, they were cleaned using deionized water. The seeds were sown in the experimental units (2 x 2 m of each unit) on the hills (3 seeds/hill) 30 cm apart, on March 15^th^, in the 2021 and 2022 seasons. There were four rows, 60 cm apart in each unit. and seven hills in every row. Thus, every experimental unit contained 28 hills. As soon as the seeds were sown, the experimental area was watered. A surface system of irrigation in the Experimental Farm was utilized using water from the Nile, which had a pH of 7.33 and an EC of 0.35 dsm^-1^. After full germination the seedlings (12 cm in height) were thinned (one seedling/hill) on first May, in the two seasons. The agricultural practices before seed sowing and after the germination of seeds (edging and leveling the soil in the used area, weeding, and insectiside and pestiside control) were done when needed.

### 2.3 The fertilizers used

#### 2.3.1 Nitrogen, phosphorus, and potassium fertilizers

The N, P, and K fertilizers at levels of 300, 200, and 100 kg/fed — ammonium sulfate (20.5% N), calcium super phosphate (15.5% P3O5), and potassium sulfate (48% K_2_O), were utilized in succession as a recommended dose (as per Ministry of Agriculture, Egypt). Calcium super phosphate was applied as one application during soil preparation. The amounts of nitrogen and potassium fertilizers were applied in four equal doses, the first dose was on second May after the thinning, the second dose was on first June, the 3^rd^ addition was 15 days after the first cut, and the 4^th^ dose was done 30 days after the 3^rd^ addition, during the experimental seasons.

#### 2.3.2 Nanoparticles

Nanoparticles of zinc oxide (ZnO NPs) at 1 and 1.5 g/L, according to Prasad et al. (2012) and Pirzad and Barin (2018). The nanoparticles of silicon (SiO_2_ NPs) at 0.1 and 0.2 g/L, as per Adhikari et al. (2013). The used nanoparticles were used as foliar spraying. Zinc oxide nanoparticles and silicon oxide nanoparticles were applied thrice. The first and second applications were sprayed 3 and 33 days after thinning of the sage seedlings and the 3^rd^ spray 20 days after the first cut. A powder of the NPs was dissolved in deionized water.

##### 2.3.2.1 Synthesis of NPs of ZnO and SiO_2_

**Figure 1:**
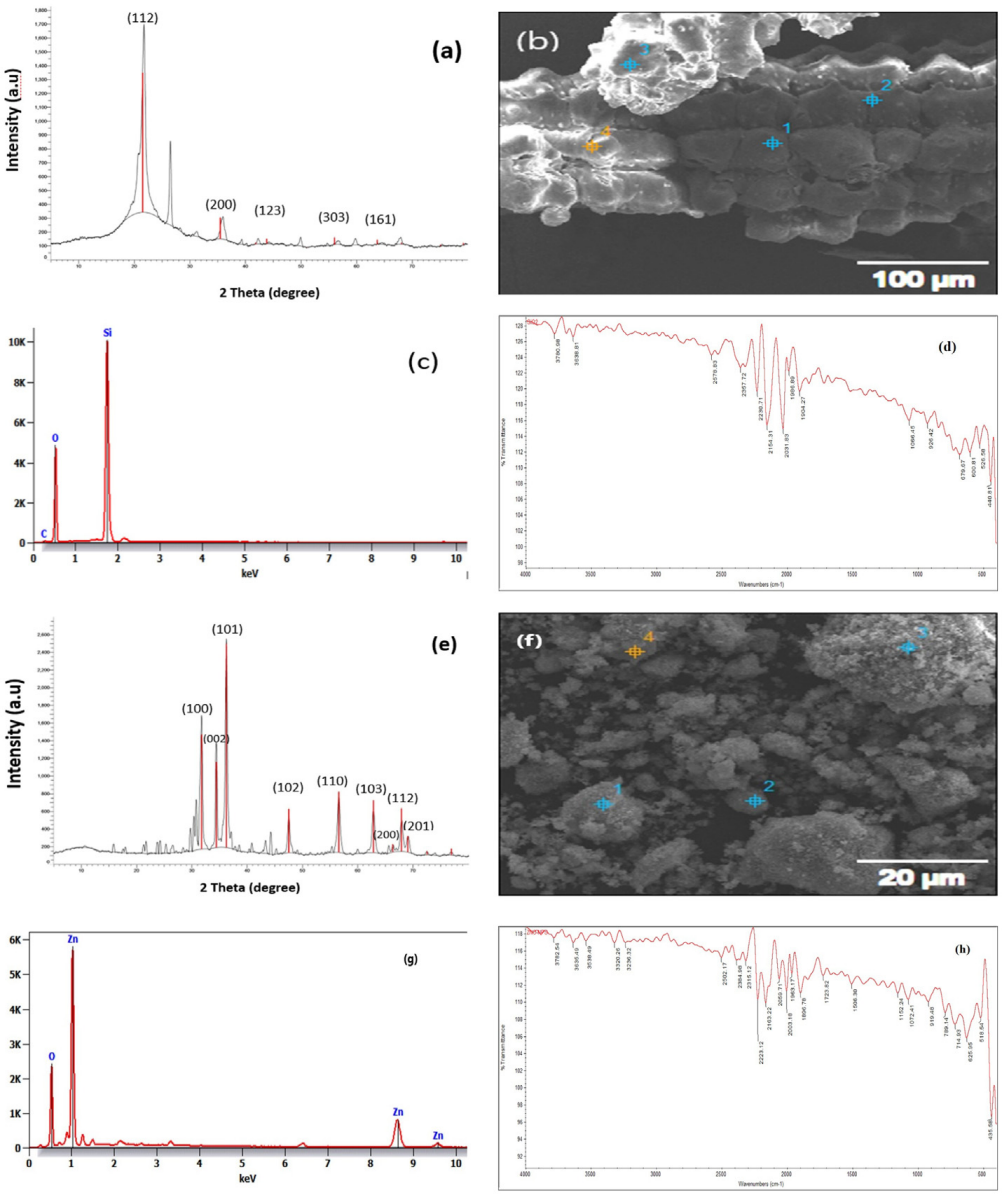
**(A)** XRD of SiO_2_ NPs; **(B)** SEM of SiO_2_ NPs; **(C)** ADX of SiO_2_ NPs; **(D)** FTIR of SiO_2_ NPs; **(E)** XRD of ZnO NPs; **(F)** SEM of ZnO NPs; **(G)** ADX of ZnO NPs; and **(H)** FTIR of ZnO NPs.

The ZnO nanoparticles were produced according to Ali et al. (2017). In brief, 50 mL of 2 m NaOH and 100 mL of 1 mM Zn (CH3COO)2. 2H_2_O were combined dropwise and stirred continuously for two hours. The white precipitate was collected using centrifugation (model 58 10r, Eppendorf Corporation, Hamburg, Germany) at 9508 rpm, for 5 minutes, at room temperature (25 ± 2°C). To eliminate the impurities, it was rinsed thrice with distilled water. The ZnO NPs were dried at 60°C for the entire night in a drying incubator (Thomas Scientific, Swedesboro, NJ, USA). The sol-gel procedure described by Hench and West (1990) was employed to prepare SiO_2_ NPs. Thirty-five milliliters of water and 65 milliliters of 100% alcohol were combined and stirred mechanically for five minutes. The preceding ethanol/water solution was then mixed with 25 mL of tetraethyl orthosilicate (TEOS) dropwise and allowed to sit at room temperature for 60 minutes, with mechanical stirring. In order to achieve this, an ammonia solution was gradually added until the gel’s full formation was determined, for which the sol–gel method was used to record that the solution had transformed into a gel. The gel that had formed was subjected to two hours of ultra-centrifugation at 7000 rpm. Ultimately, the precipitated wet gel was gathered and subjected to three rounds of distilled water washings to eliminate any unwanted chemical or any chemical that had not reacted (TEOS). The moist gel was left for calcination at 700° C for 5–7 hours in an Empyrean PAN analytical X-ray diffractometer with Bragg-Brentano geometry and Cu Ka radiation (R = 1.54A). The powder patterns of the silicon oxide and zinc oxide nanoparticles were registered. The step scan spanned the angular range of 20 to 80 at a step of 0.02. The size of the crystallite was calculated using the Scherrer equation (D = Kh/B cos B). The crystallite size (D), the X-ray radiation wave length (h), (K) the constant (0.94), the line width at half the peak’s greatest intensity (B), and the diffraction angle (QB), are all represented in this equation. (**Figures 1–D**) show SEM, FTIR, XRD, EDX of the SiO_2_ NPs and (**Figures 1E–H**) show SEM, FTIR, XRD, EDX of ZnO NPs.

The chemical bonding nature of the nanoparticles was assessed using the Fourier-transform infrared (FTIR) absorption spectroscopy (THERMO NICLOT, 50.UK). The powder’s shape and elemental content were investigated using scanning electron microscopy (SEM; JEOL, JSM-6360LA, Tokyo, Japan) during the removal process (Naddaf et al., 2020). One method for describing crystalline materials was X-ray diffraction XRD device (Panalytical Emperian, Istanbul, Turkey). It offered details on phases, preferred crystal orientations (texture), and additional structural characteristics, such as, average grain size, strain, crystallinity, and crystal defects (Pandey et al., 2021). Energy-dispersive X-ray spectroscopy (EDX; JEOL JSM-5300, USA) was a method for elemental analysis related to electron microscopy that used the production of distinctive X-rays. to identify the elements that were present in the samples (Scimeca et al., 2018).

The specification, physical and chemical properties, and acute toxicity of the used ZnO NPs and SiO_2_ NPs are presented in (**Table 2**).

**Table 2:** The specification, physical and chemical properties, and acute toxicity of the used ZnO NPs and SiO_2_ NPs. .

	Silica oxide nanoparticles	Zinc oxide nanoparticles
	Specification	
Appearance	white powder	white powder
Average Particle size	15 ± 10 nm	20nm
Morphology	Spherical.	Spherical.
Surface area	109.356 m^2^/g	2.7534 m^2^/g
Average pore radius	3.53198e+01Å	40.5965 nm
Total pore volume	1.931e-02 cc/g	0.042062 cm^3^/g
Chemical Composition	silicon = 46.83%Oxygen 53.33%	Zn= 80.34%O= 19.6
Physical and chemical properties		
**Odor**	odorless	odorless
**Density**	at 20 c° (1.973 g/cm^3^)	at 20 c° (5.6 g/cm^3^)
**Melting Point**	1600-1728 c°	1957c°
**Boiling Point**	2230c°	2360 c°
**Solubility:**	insoluble in water	insoluble
**Acute Toxicity**	Inhalation human LD50 = 3000mg/kg Intravenous rat LD50 = 90mg/kg Intravenous mouse LD50 = 40mg/kg Oral rat LD50>3000mg/kg Dermal rabbit LD50>5000 mg/kg	The lethal dose 50 (LD50) of intravenously administered =0.3 mg/kg in mice TheLD50of intratracheal instillation= 493.85 µg/kg in mice.

#### 2.3.3 Date pollen extract

In Damanhur city, Elbehyra Governorate, Egypt, pollen from Egyptian date palms (*Phoenix dactylifera* L.) was collected in the last week of March, in the seasons of 2021 and 2022, during the male inflorescence cover opening. The pollen extract was prepared according to Nagai et al. (2002) method, with some changes: For one hour, 0.1g of pollen was added to 10.0 mL of deionized water. Next, the mixture was centrifuged (Sigma 3-18KS, SIGMA Laborzentrifugen GmbH, Osterode am Harz, Germany) at 5000 rpm for 10 minutes at a temperature of 20°C. The ultrasonic probe, VCX 750 (SONICS & MATERIALS, INC., Newtown, CT, USA), was used to sonicate the mixture for 30 seconds at a frequency of 6 KHZ. In every experiment, the resultant supernatant was used as a water pollen extract. Distilled water was then added to complete the extract, to obtain the used levels (15 and 25g/L), as per Abou-Sreea and Yassen (2016). Date pollen extract was sprayed thrice. The first and second applications were carried out 2 and 31 days after thinning of the sage seedlings, and the 3rd application was performed 22 days after the first cut.

### 2.4 The used fertilizer types and fertilization treatments

In this experiment, ten fertilizer applications were performed (**Table 3**).

**Table 3:** The used fertilization treatments.

Treatments No.	The fertilization treatments
T1	100% NPK (recommended dose) as a control
T2	75% NPK + 15 g/L date pollen extract (DPE)
T3	50% NPK + 25 g/L DPE
T4	75% NPK + 0.1 g/L SiO_2_ NPs
T5	50% NPK + 0.2 g/L SiO_2_ NPs
T6	75% NPK + 1 g/L ZnO NPs
T7	50% NPK + 1.5 g/L ZnO NPs
T8	50% NPK + 25 g/L DPE + 0.2 g/L SiO_2_ NPS
T9	50% NPK + 15 g/L DPE + 1.0 g/L ZnO NPs + 0.1 g/L SiO_2_ NPs
T10	25% NPK + 25 g/L DPE + 1.5 g/L ZnO NPs + 0.2 g/L SiO_2_ NPs.

### 2.5 Experimental layout

In this investigation, a randomized complete block design (RCPD) was used. Three replicates of the experiment were done with ten treatments in each replicate (Snedecor and Cochran, 1989). Each treatment contained three experimental units in one way ANOVA.

### 2.6 Data recorded

#### 2.6.1 Growth parameters

The first and second cuts of sage were done on July first and November first of the 2021 and 2022 seasons, with five plants randomly harvested from each experimental unit. The vegetative characteristics were measured, as the average of the two cuts of each season: Plant height (cm) was estimated from the soil surface to the plant’s top; fresh weight and constant air dry weights of aerial organs/plant (g) and chlorophyll index of the fifth leaf from the top of the branch was estimated using SPAD units, measured with a Minolta SPAD chlorophyll meter model-502 (Yadava, 1986).

#### 2.6.2 Essential oil percentage and yield

Air-dried sage herb samples at constant weight (50 g/sample) were used for hydro distillation with sterile water, 1 L for 3 hours, in a Clevenger-type apparatus. The collected essential oil (EO) was dried over anhydrous sodium sulfate and kept for later use at 4°C (Elmsellem et al., 2019). Where,


$$EO\%=\frac{oil\;volume\;in\;graduated\;tub\;}{\;\;plant\;sample\;weight\;}\times 100\;$$

Plant essential oil  (mL)=EO%X plant dry weight

Where the percentage and yield of the essential oil/plant were determined as the average of the two cuts of each season.

#### 2.6.3 Gas chromatography/mass spectrometry analysis of oil

For identifying the EO compounds during the first cut of the second season, gas chromatography/mass spectrometry (GC–MS) analysis was utilized. The sample was obtained and filtered, to ensure that it wouldn’t impact the column, and then one microliter of the sample was inserted into the GC. An Agilent 6890 N gas chromatograph with a capillary column DB-5 MS (30 m × 250 μm × 0.25 μm) from Agilent Technologies, USA, and a 5975 B mass selective detector spectrometer from the same firm were attached to the gas chromatograph. A split mode maintained the front inlet at 250°C. This was the temperature program: 60°C was the starting point and was held for two minutes; after that, it was programmed to reach 120°C at a pace of 6°C per minute, and was kept for two minutes; finally, it was designed to reach 230°C at a rate of 4°C per minute, and was maintained for five minutes. The split injection flow rate was one milliliter per minute. As a carrier gas, 1.0 mL of helium per minute was employed. An ionization voltage of 80 eV was employed when using the MS detector in the EI mode. The temperature of the ion source was 230°C. It reached 280°C in the transfer line. The mass range (m/z) 30–1000 was covered by the spectrum collection. The retention indices were computed using the retention periods of n-alkanes, C6–C26, that were injected under identical chromatographic conditions. They were identified by using the Nits 08.L library of essential oil compounds, by comparing the mass spectra and relative retention indices of the volatile components.

#### 2.6.4 Total phenol compounds and antioxidant activity

The dried leaf samples from the two cuts in the second season were the only ones used to assess the total phenol compounds (TPC) and antioxidant activity (AOA). The air-dried leaves were ground and soaked in methanol. The mixture was filtered after 24 hours, and the amount of total phenols was measured using the filtrate. With external calibration using gallic acid and the Folin–Ciocalteu reagent, the amount of TPC in the crude extracts was ascertained. To summarize, 0.2 milliliters of extract solution and 0.2 milliliters of the Folin-Ciocalteu reagent were added, and their contents were properly mixed (Singleton et al., 1999). One milliliter of 15% Na2CO3 was added after four minutes, and the mixture was then left to stand at room temperature for two hours. A Spectro (Thermo Fisher Scientific, Waltham, MA, USA model 4001/4) spectrophotometer was used to detect the absorbance at 760 nm. Using an equation derived from the gallic acid calibration curve, the concentration of TPC was determined as a milligram of gallic acid equivalent/g dry weight (D.W). The results were an average of the three separate measurements that were made of the TPC, in each of the fractions (Martínez-Esplá et al., 2014).

The 2,2-diphenyl-1-picryl hydrazyl (DPPH) assay was used to evaluate the antioxidative capacity of dry leaves (Binsan et al., 2008), with a tiny alteration. To a 0.1% protein solution (in 5 mM poly (1,4-butylene succinate) (PBS) buffer, pH 7.2), 0.15 mM of 2,2-diphenyl-1-picryl hydra Zyl (DPPH), in 95% ethanol, was added, in a ratio of 1:1 (v/v). The blend was combined and allowed to sit at room temperature for half an hour, in the dark. A spectrophotometer (Helios Gamma; Thermos Fisher Scientific) was used to measure the absorbance of the resultant solution at 517 nm. The preparation of the blank was identical to that of the sample, with the exception that the 5 mM PBS buffer (pH 7.2) was utilized. Trolox was used to produce the calibration curve in the 12.5–100 μM range. The Trolox equivalent (TE)/mg of dry leaves was the unit of expression for the activity. The data of TPC and AOA have been calculated as an average of the two cuts in the 2022 season.

### 2.7 Leaf chemical composition

Leaves samples of the two cuts in 2^nd^ season were dried in the oven at 72°C for 36 h and ground to obtain a homogenous powder in a metal-free mill (Ika-Werke, M 20 Germany). Concentrated sulfuric acid (95%, 5 mL) was added to the 0.2 g sample, then a sand hotplate was used to heat the mixture for 10 min. After that, 0.5 mL of perchloric acid was dropwise, and heating was continued to obtain a clear solution. The solution was left to cool, and filtered, then it was diluted to 50mL (Evenhuis and de Waard, 1980). The measurements of N, P, and K percentages were estimated by a modified micro- Kjeldal method (Chemists, 1990), spectrophotometer (GT 80+, UK) (Murphy and Riley, 1962), and an atomic absorption spectrophotometer (Avanta E; GBC, Victoria, Australia) (Cottenie et al., 1982), respectively. Silicon, Zn and total carbohydrates percentages were measured using the techniques of Hogendorp et al. (2012); Jackson (1973), and Herbert et al. (1971), consecutively. Data on leaf chemical composition were calculated as the average of the two cuts in 2022 seasons.

### 2.8 Statistical analysis

The data underwent analysis of variance using the SAS software (Version 6.12; SAS Institute Inc., Cary, NC, USA). The mean separations ( ± SE) were computed by a one-way ANOVA, with significance established at *p* ≤ 0.05, by using the Duncan’s multiple range test (DMRT).

## 3 Results

### 3.1 Vegetative growth characters

**Figure 2:**
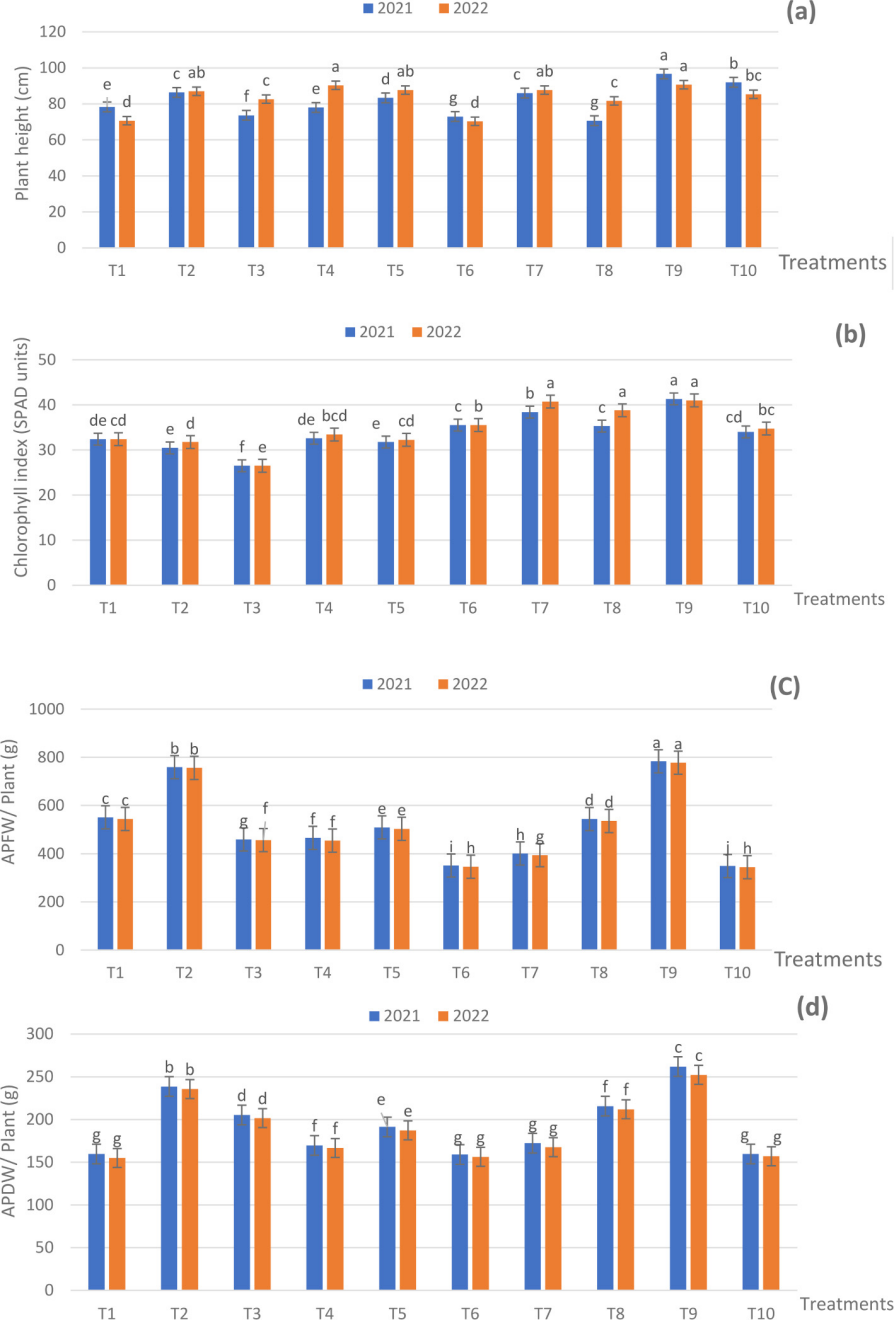
Effect of fertilization treatments during 2021 and 2022 seasons on, **(A)** plant height; **(B)** chlorophyll index; **(C)** APFW; and **(D)** APDW. The means have similar letters within the figures that denote non-significance (*p* ≤ 05), according to Duncan’s multiple range test. T1 - 100% NPK (recommended dose) as a control, T2 - 75% NPK + 15g/L date pollen extract (DPE), T3 - 50% NPK + 25 g/L DPE, T4 - 75% NPK + 0.1 g/L SiO_2_ NPs, T5 - 50%NPK + 0.2 g/L SiO_2_ NPs, T6 - 75% NPK + 1 g/L ZnO NPs, T7 - 50% NPK + 1.5 g/L ZnO NPs, T8 - 50% NPK + 25 g/L DPE + 0.2 g/L SiO_2_ NPS, T9 - 50% NPK + 15 g/L DPE + 1.0 g/L ZnO NPs + 0.1 g/L SiO_2_ NPs, and T10 - 25% NPK + 25g/L DPE + 1.5 g/L ZnO NPs + 0.2 g/L SiO_2_ NPs.

During the experimental seasons, treatments combining 75%, 50%, and 25% NPK RD with ZnO NPs, SiO_2_ NPs, or DPE at various concentrations notably increased the height of sage plants compared to the control (NPK RD) with some exceptions (**Figure 2A**). Additionally, the tallest plants were those that received T9 in the two seasons. This treatment resulted in a height of 96.66 and 90.66 cm against 78.33 and 70.66 cm for the control in both seasons, respectively. It is obvious that the variations between the used fertilization treatments were unable to arrive at the significant level (*p* ≤ 0.05) in some cases.

With regard to the chlorophyll index, the majority of fertilization applications exhibited positive effects on the SPAD values over the respective control (**Figure 2B**). The highest significant SPAD values (*p* ≤ 0.05) were recorded for plants that received T9 of 41.33 and 41.00 SPAD units against 32.40 and 32.40 SPAD units for the control in the two seasons, respectively.

The findings of the aerial part fresh weight (APFW) and aerial part dry weight (APDW) of the sage plant clearly showed that fertilization treatments had differently affected the APFW and APDW (**Figures 2C, D**). Whereas, in the two seasons the treatment of T9 resulted in a higher significant APFW of 783.83 and 777.66 g/plant and APDW of 262.00 and 252.33 g/plant, consecutively Furthermore, the fertilization treatments significantly increased the APFW and APDW compared to NPK RD in the two seasons, with some exceptions; even as the control gave an APFW of 551.00 and 544.00 g/plant and APDW of 159.66 and 155.00 g/plant in the two seasons, in succession. The differences among the used applications reached the significant level in a majority of cases during the experimental seasons. The application of ZnO and SiO_2_ nanoparticles, along with DPE, has shown positive effects on vegetative growth parameters, particularly when reducing NPK by 100%, with T9 showing the most notable improvement.

### 3.2 Essential oil productivity

**Figure 3:**
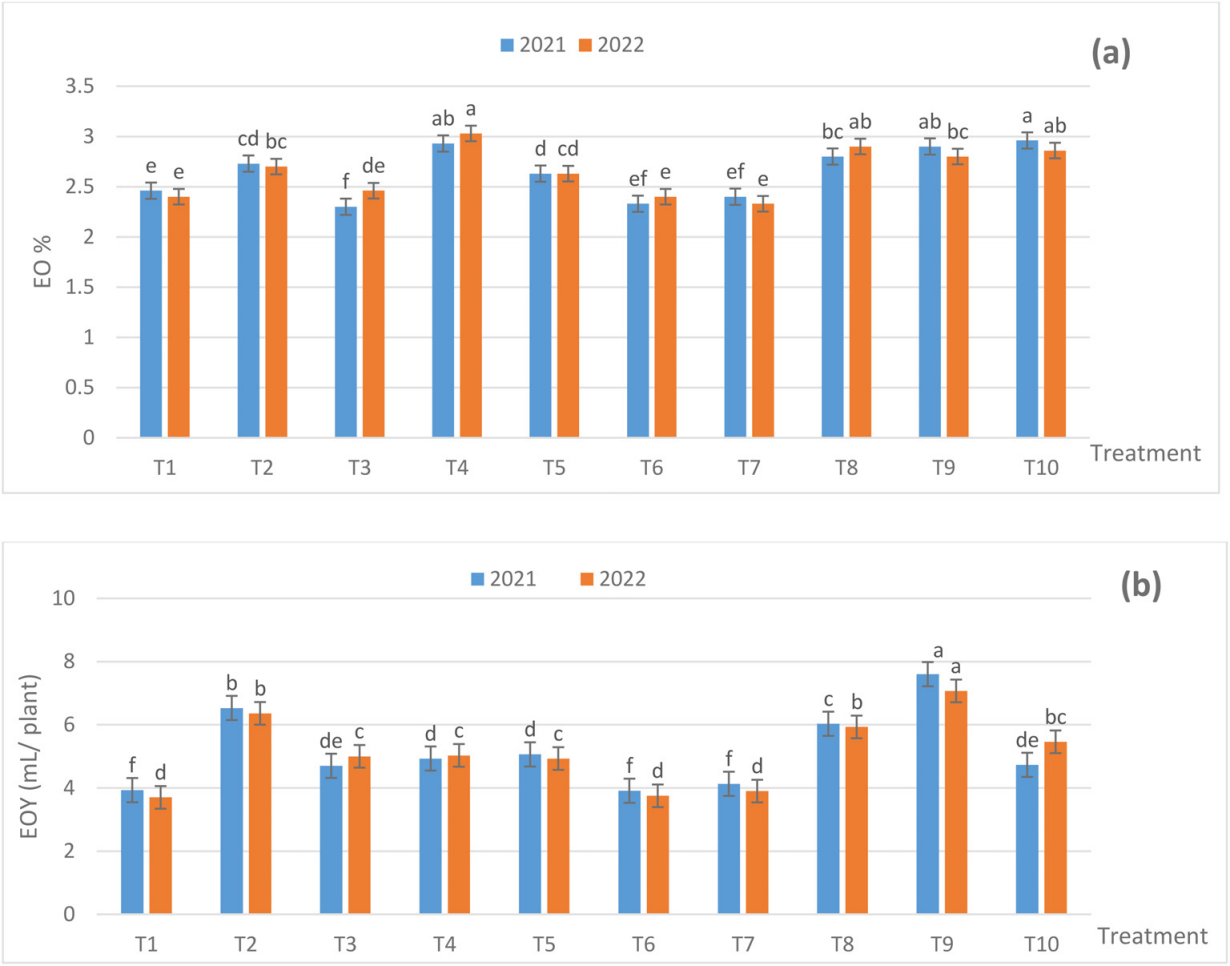
Effect of fertilization treatments during 2021 and 2022 seasons on **(A)** EO% and **(B)** EOY (ml/plant). The Duncan’s multiple range test indicates that there was no significant difference (*p* ≤ 0.05) among the means, as seen in the figures that are followed by the same letters. T1- 100% NPK (recommended dose) as a control, T2 - 75% NPK + 15 g/L date pollen extract (DPE), T3 - 50% NPK + 25 g/L DPE, T4 - 75% NPK + 0.1 g/L SiO_2_ NPs, T5 - 50% NPK + 0.2 g/L SiO_2_ NPs, T6 - 75% NPK + 1 g/L ZnO NPs, T7 - 50% NPK + 1.5 g/L ZnO NPs, T8 - 50% NPK + 25 g/L DPE + 0.2 g/L SiO_2_ NPS, T9 - 50% NPK + 15 g/L DPE + 1.0 g/L ZnO NPs + 0.1 g/L SiO_2_ NPs, and T10 - 25% NPK + 25g/L DPE + 1.5 g/L ZnO NPs + 0.2 g/L SiO_2_ NPs.

Fertilization treatments influenced EO% and EO yield (EOY) (based on herb dry weight) (**Figures 3A, B**). All treatments caused significant increases in EO% and EOY in relation to 100% NPK (NPK RD) throughout the experimental duration, except for T3 in the first season and T6 and T7 during the two seasons in case of EO%. The maximum significant EO% reached 2.96% for the T10 treatment in the first season. Although, in the second season the EO% reached 3.03% for the T4 treatment. At the same time, the T1 plants had 2.33% and 2.40% EO in both the seasons, consecutively. Moreover, the T9-treated plants produced a maximum significant EOY of 7.60 and 7.07 mL/plant in both the seasons, respectively. The EOY in the control plants reached 3.93 and 3.70 mL/plant in both the seasons, successively. Also, the impact of the used fertilization treatments on EO% and EOY reached the significant level (*p* ≤ 0.05) among most of the applications used, in the both seasons. It is obviouse from data of EO% and EOY that the treatments contant ZnO NPs, SiO_2_ NPS and DPE had positive effect on such traits or decreasing NPK RD.

GC-MS analysis of sage EO (**Table 4**) showed the identification of 58 compounds distributed among the 10 used treatments. T8 resulted in the maximum EO compounds of 32, while T10 showed the least EO compounds of 11 compounds. The other treatments produced intermediate numbers of compounds. Specifically, T3, T7, and T8 exhibited compound numbers in EO that were higher than those observed with 100% NPK. In contrast, the remaining treatments resulted in compound numbers in EO that were lower than those with 100% NPK. The total compounds represented 98.60% for the control to 99.99% for T4, for the EO of sage. Oxygenated hydrocarbons ranged from 79.05% in T9 to 88.94% in T6 of the total EO chemical structure. These were followed by sesquiterpene hydrocarbons reaching 9.38% for T6 to 16.29% for T9. Then, monoterpene hydrocarbons had the lowest content of sage EO ranged from 0.00% for T8 to 3.61% for T2. The analysis exhibited that the dominant constituents (> 3%) were thujone (9.96% in T8 to 29.86% in T6), (+)-2-bornanone/(15)- (-1- camphor (4.61% in T8 to 15.77% in T6), manool (6.84% in T7 to 14.23% in T10), ledol (8.45% in T8 to 13.78% in T4 and T9), cineol (6.34% in T8 to 12.77% in T1), Estragole (0.87% in T7 to 11.86% in T8), Endo-Borneal (4.40% in T5 to 8.96% in T3), caryophyllene (3.03% in T6 to 8.76% in T8), Humulene (4.76% in T8 to 7.43% in T1) and caryophyllene oxide (3.06% in T8 to 5.88% in T4). The EO analysis revealed that nine compounds were observed in all treatments, while some constituents were noticed in one or two treatments only.

**Table 4:** EO composition of *Salvia officinalis* as affected by the utilizations used at first cut in the second season.

Compound name (%)		Treatments									
		Control (T1)	T2	T3	T4	T5	T6	T7	T8	T9	T10
1	Trilinolein	0.21	–	–	–	–	–	–	–	–	–
2	Beta-pinen	1.09	0.52	–	–	0.43	–	1.51	–	0.53	–
3	Butyl	0.28	–	–	–		–	–	–	–	–
4	P-Cymene	0.46	0.80	0.63	0.65	0.70	–	0.67	–	0.78	–
5	Cineole	12.77	12.7	12.22	11.12	9.91	10.8	10.41	6.34	12.71	10.82
6	Thujone	24.96	29.21	21.14	25.00	26.17	29.86	21.35	9.96	22.53	26.6
7	(+)-2-bornanone/(1S)- (-)-Camphor	8.45	11.28	12.04	8.74	12.66	15.77	10.35	4.61	10.65	7.02
8	Endo-Borneol	5.49	5.54	8.96	4.97	4.40	4.53	6.39	5.46	5.25	6.09
9	Estragole	1.55	–	1.84	2.15	0.87	–	5.30	11.5	–	–
10	Á-copaene	0.23	–	0.28	0.24	0.22	–	0.57	0.46	0.35	–
11	Caryophyllene	5.22	4.79	5.37	3.80	5.13	3.03	5.53	8.76	7.03	7.79
12	Humulene	7.43	7.24	6.36	6.19	6.32	6.35	6.09	4.76	7.01	6.69
13	À-ylangene	0.31	–	–	0.26	–	–	0.36	–	0.20	–
14	Caryophyllene oxide	3.36	3.32	4.55	5.88	3.90	4.42	5.28	3.06	3.89	5.52
15	Ledol/Viridiflorol	13.69	9.61	11.71	13.73	12.17	11.08	13.52	8.45	13.73	11.96
16	Bornyl acetate	1.09	1.76	1.84	1.56	1.15		1.50		1.09	1.77
17	Caryophylla-4 (12),8 (13)-dien-5à-ol	0.65	0.25	0.21	0.44	0.25	–	–	0.95	0.76	–
18	Labda-8 (20),14-dien-13-ol, (13R)-; Manool	10.41	7.55	8.77	12.33	12.52	10.17	6.84	9.50	8.93	14.23
19	Aromadendrene	0.44	1.12	0.32	0.50	0.56	–	0.27	0.77	0.81	–
20	Á-Pinene	–	2.12	1.77	1.41	1.46	–	0.39	–	2.05	–
21	Cis-ocimene	–	0.17	–	–	–	–	–	–	0.20	–
22	Guaia-1 (10),11-diene	–	0.51	–	–	–	–	–	–	–	–
23	Á-cadinene	0.51	0.30	0.43	0.41	0.35	–	–	–	–	–
24	Linalool	–	–	0.34	0.21	–	–	0.33	–	–	–
25	Cis-à-Bergamotene	–	–	0.27	–	–	–	–	–	–	–
26	LEDEN	–	0.51	0.30	–	–	–	–	–	0.34	–
27	Cis-à-Bisabolene	–	–	0.20	–	–	–	–	–	–	–
28	Methyleugenol	–	–	–	–	–	–	0.83	–	–	–
29	Retinal	–	–	–	–	–	0.60	–	–	–	–
30	Isobornyl acetate	–	–	–	–	–	1.24	–	–	–	–
31	À-acorenol	–	–	–	–	–	–	0.70	–	–	–
32	Eugenol	–	–	–	–	–	–	0.56	–	–	–
33	Á-Guaiene	–	–	–	–	–	–	0.19	–	–	–
34	(-)-Spathulenol	–	0.38	–	–	–	–	0.27	0.97	–	–
35	Diethyl Phthalate	–	–	–	–	–	–	0.29	–	–	–
36	9-OCTADECENOIC ACID (Z)-	–	–	–	–	–	–	–	2.72	–	–
37	TETRADECANE, 1-CHLORO	–	–	–	–	–	–	–	0.74	–	–
38	1-Hexadecanol, 2-methyl-		–	–	–	–	–	–	3.10	–	–
39	3-OCTADECYNE	–	–	–	–	–	–	–	2.60	–	–
40	Isobornyl thiocyanoacetate	–	–	–	–	–	–	–	2.24	–	–
41	Trans-Sesquisabinene hydrate	–	–	–	–	–	–	–	0.82	–	–
42	10-Heptadecen-8-ynoic acid, methylEster, (E)-	–	–	–	–	–	–	–	0.79	–	–
43	1,3-benzodioxole, 5-[[2- (2-butoxyethoxy)etho Xy]methyl]-6-propyl-	–	–	–	–	–	–	–	0.50	–	–
44	1-Heptatriacotanol	–	–	–	–	–	–	–	1.35	–	–
45	9-octadecenoic acid (z)-	–	–	–	–	–	–	–	1.65	–	–
46	Thunbergol	–	–	–	–	–	–	–	2.01	–	–
47	Patchouli alcohol	–	–	–	–	–	–	–	1.67	–	–
48	EPISTEPHAMIERSINE	–	–	–	–	–	–	–	0.50	–	–
49	7-Hydroxy-6,9a-dimethyl-3-methylene-decahydro-azuleno[4,5-b]furan-2,9-dione	–	–	–	–	–	–	–	0.54	–	–
50	(2-Aminocyclohexyl)-phenyl-methanol	–	–	–	–	–	–	–	0.48	–	–
51	Cyclopropanedodecanoic acid, 2-octyl-, methyl ester		–	–	–	–	–	–	1.08	–	–
52	Galactopyranose, 5TMS derivative	–	–	–	–	–	–	–	0.81	–	–
53	3-Buten-2-ol, 4- (2,6,6 trimethyl-2-cyclohexen-1-yl)-, (3E)-	–	–	–	–	–	1.91	–	–	–	–
54	3-METHYL-5- (2,6,6-TRIMETHYL-1 -CYCLOHEXEN-1-YL)-1-PENTYN-3-OL	–	–	–	–	0.77	–	–	–	–	–
55	CYCLOHEXENE, 1,5,5-TRIMETHYL-6-METHYLENE	–	–	0.14	–	–	–	–	–	–	–
56	TETRADECANE, 1-CHLORO-	–	–	–	–	–	–	–	0.74	–	–
57	Doconexent	–	–	–	0.40	–	–	–	–	–	–
58	Trans-p-mentha-1 (7),8-dien-2-ol	–	–	–	–	–	–	–	–	–	0.49
	Total Compounds (%)	98.60	99.68	99.69	99.99	99.94	99.76	99.50	99.89	98.84	98.98
	Monoterpene Hydrocarbons (%)	1.09	3.61	0.63	2.06	2.59	1.44	2.66	0.00	3.50	1.33
	Sesquiterpene Hydrocarbons (%)	14.5	13.71	14.29	11.44	12.58	9.38	13.81	15.57	16.29	14.48
	Oxygenated Hydrocarbons (%)	83.01	82.36	84.77	86.49	84.77	88.94	83.03	84.32	79.05	83.17
	Number of Compounds (%)	20	20	22	20	19	12	24	32	19	11

T1- 100% NPK (recommended dose) as a control, T2- 75% NPK +15g/L date pollen extract (DPE), T3- 50% NPK +25 g/L DPE, T4- 75% NPK + 0.1 g/L SiO_2_ NPs, T5-50%NPK + 0.2 g/L SiO_2_ NPs, T6- 75% NPK + 1 g/L ZnO NPs, T7-50% NPK + 1.5 g/L ZnO NPs, T8- 50% NPK + 25 g/L DPE + 0.2 g/L SiO_2_ NPS, T9- 50% NPK + 15 g/L DPE + 1.0 g/L ZnO NPs+ 0.1 g/L SiO_2_ NPs, and T10- 25% NPK +25g/L DPE+ 1.5 g/L ZnO NPs + 0.2 g/L SiO_2_ NPs.

### 3.3 Total phenol compounds and antioxidant activity

**Figure 4:**
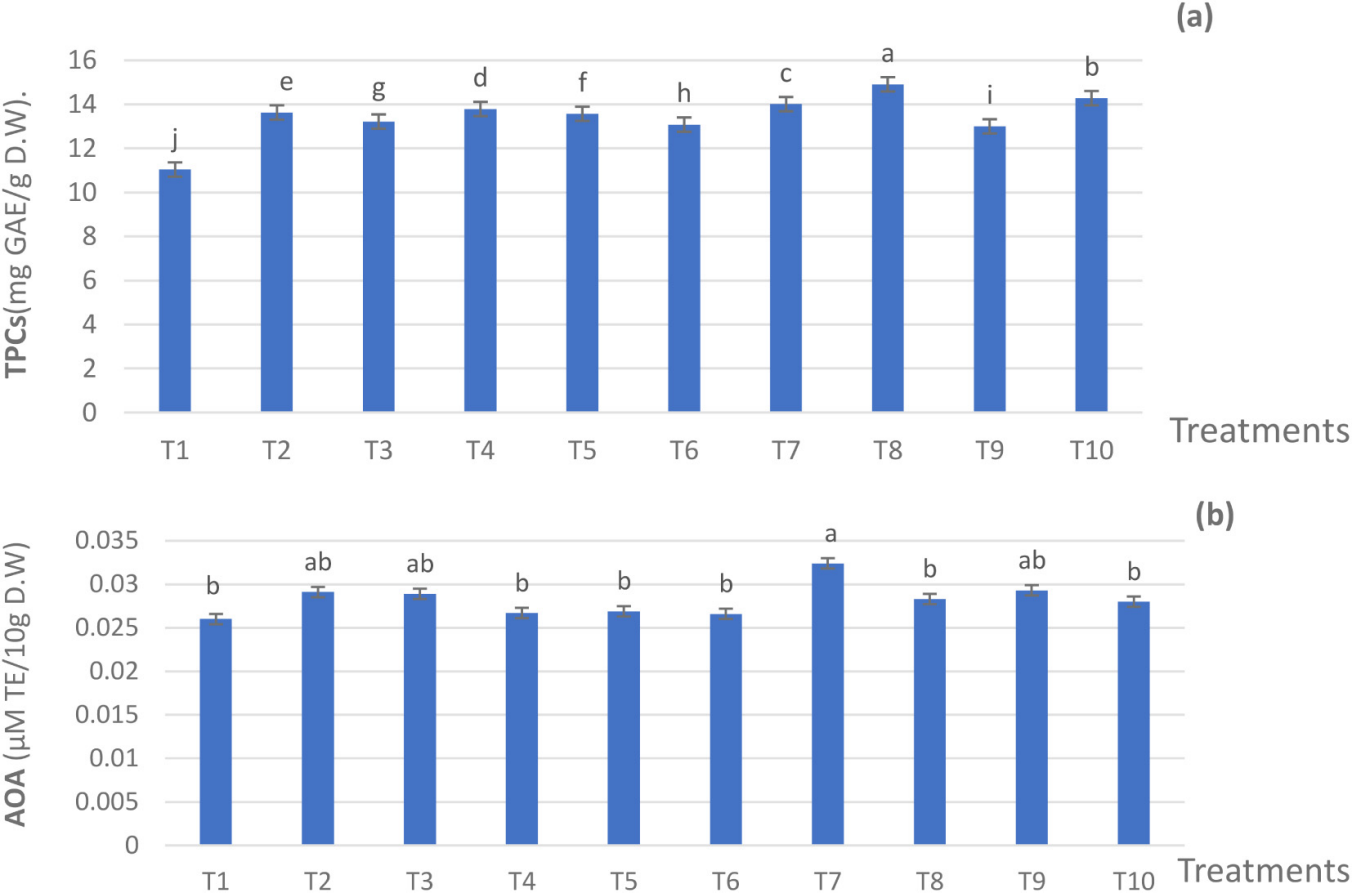
Effect of the fertilization treatments during the 2022 season on **(A)** TPC and **(B)** AOA. The Duncan’s multiple range test indicates that there is no significant difference (*p* ≤ 0.05) among the means in the figures that are followed by the same letters. T1 - 100% NPK (recommended dose) as a control, T2 - 75% NPK + 15 g/L date pollen extract (DPE), T3 - 50% NPK + 25 g/L DPE, T4 - 75% NPK + 0.1 g/L SiO_2_ NPs, T5 - 50%NPK + 0.2 g/L SiO_2_ NPs, T6 - 75% NPK + 1 g/L ZnO NPs, T7 - 50% NPK + 1.5 g/L ZnO NPs, T8 - 50% NPK + 25 g/L DPE + 0.2 g/L SiO_2_ NPS, T9 - 50% NPK + 15 g/L DPE + 1.0 g/L ZnO NPs + 0.1 g/L SiO_2_ NPs, and T10 - 25% NPK + 25 g/L DPE + 1.5 g/L ZnO NPs + 0.2 g/L SiO_2_ NPs.

All fertilization treatments of 75,50, and 25% NPK RD added to ZnO NPs, SiO_2_ NPs, and DPE at various levels, significantly promoted TPCs, as compared to T1 (**Figure 4A**). Also, the differences among the mean values of all treatments were significant (*p* ≤ 0.05). Among all the treatments, the significant maximum value of TPCs was found in plants that received T8, resulting in 14.91 mg GAE/g D.W compared to 11.04 mg GAE/g D.W for T1.The other treatments resulted in intermediate values of TPCs. Even as the mean values of AOA (**Figure 4B**) ranged from 0.0260 micromole Trolox equivalent (**µ**MTE)/10g D.W) for T1 to 0.0324 µM TE/10g for T7. It was noticed that the AOA values of the used applications were close. Thus, AOA was positively improved by applying ZnO nanoparticles, SiO_2_ nanoparticles, and DPE in combination with 75%, 50%, and 25% of the NPK, compared to the 100% NPK treatment.

### 3.4 Leaf chemical composition

To estimate the impact of different fertilization treatments on leaf chemical composition, the levels of nitrogen (N), phosphorus (P), potassium (K), zinc (Zn), silicon (Si), and total carbohydrates (%) in the leaves were measured (**Table 5**). The data indicate that the applied treatments had significantly different effects on leaf chemical composition. Specifically, the differences in the mean values of N, P, K, Zn, Si, and total carbohydrates among the various fertilization treatments were significant in most cases. The highest significant values were recorded as follows: N at 2.06% for both T1 and T2, P at 0.86% for T1, K at 3.03% for T9, Zn at 0.16% for T7, Si at 0.02336% for T10, and total carbohydrates at 1.73% for T7. Conversely, the lowest significant values were N at 1.11% for T8, P at 0.35% for T5, K at 1.61% for both T5 and T8, Zn at 0.05% for T9 and T10, Si at 0.00012% for T1, and total carbohydrates at 0.83% for T2 and T4.

**Table 5:** Impact of the fertilization treatment on *Salvia officinalis* leaf N, P, K Zn, Si and total carbohydrate % in 2022 season.

Fertilization treatments	N%	P%	K%
T1	2.06 ± 0.01a	0.86 ± 0.03a	2.09 ± 0.001e
T2	2.06± 0.01a	0.60 ± 0.01e	2.10± 0.00 d
T3	1.76± 0.01 b	0.70 ± 0.00c	2.21± 0.02 c
T4	1.30± 0.00d	0.73± 0.03 b	1.89± 0.001 e
T5	1.23± 0.01 d	0.53± 0.01 f	1.61± 0.02 f
T6	1.50± 0.01 c	0.55± 0.01 f	2.50± 0.01 b
T7	1.50 ± 0.01c	0.59± 0.01 e	2.50± 0.01 b
T8	1.11 ± 0.00e	0.63 ± 0.02d	1.61± 0.02 f
T9	1.30 ± 0.00d	0.75 ± 0.00 b	3.03± 0.06 a
T10	1.53 ± 0.01c	0.73 ± 0.01 b	2.52± 0.03 b
	Zn%	Si%	Total carbohydrate %
T1	0.07± 0.00 g	0.00010 g	1.13 ± 0.05 d
T2	0.08± 0.00ef	0.00020 g	0.86 ± 0.11 e
T3	0.07 ± 0.01 fg	0.00443 e	1.13± 0.02 d
T4	0.10± 0.00 c	0.00143 f	0.83 ± 0.05 e
T5	0.09± 0.01 cd	0.00196 f	0.93 ± 0.05 e
T6	0.12± 0.00 b	0.00183 f	1.23± 0.06 cd
T7	0.16± 0.00 a	0.00873 c	1.73 ± 0.10 a
T8	0.08± 0.01 de	0.01406 b	1.53± 0.02 b
T9	0.05 ± 0.01 h	0.00623 d	0.93 ± 0.05 e
T10	0.05± 0.01 h	0.02336 a	1.33± 0.06 c

Means in each column followed by the such letters are not significantly different (p ≤ 0.05) by Duncan’s Multiple Range Testing.

T1- 100% NPK (recommended dose) as a control, T2- 75% NPK +15g/L date pollen extract (DPE), T3- 50% NPK +25 g/L DPE, T4- 75% NPK + 0.1 g/L SiO_2_ NPs, T5- 50% NPK + 0.2 g/L SiO_2_ NPs, T6- 75% NPK + 1 g/L ZnO NPs, T7-50% NPK + 1.5 g/L ZnO NPs, T8- 50% NPK + 25 g/L DPE + 0.2 g/L SiO_2_ NPS, T9- 50% NPK + 15 g/L DPE + 1.0 g/L ZnO NPs+ 0.1 g/L SiO_2_ NPs, and T10- 25% NPK +25g/L DPE+ 1.5 g/L ZnO NPs + 0.2 g/L SiO_2_ NPs.

The most effective fertilization treatment improved leaf chemical composition compared to the 100% NPK treatment, with the exception of nitrogen (N%) and phosphorus (P%) content.

## 4 Discussion

Climatic conditions (such as temperature, relative humidity, photoperiod, and light intensity) and soil conditions (including pH and physical and chemical properties) influence the physico-chemical properties of nanoparticles. Consequently, the study was conducted in an open field to account for these variables.

Our investigation tested the potential of combining NPs of ZnO and SiO_2,_ and DPE with 75%, 50%, and 25% NPK RD, as a means of reducing the risk associated with traditional fertilizers. The study revealed a positive effect on the reduction of NPK application through the use of ZnO NPs, SiO_2_ NPs, and DPE. This observation suggested that a balance could be achieved between 75%, 50%, and 25% NPK RD and either DPE or NPs of the used elements, leading to an increase in vegetative traits. Noreen et al. (2018) revealed that Zn has an important function in plant growth, being a principal component or a principal co-factor of several proteins, as well as enzymes. Nano zinc has a vital role in various processes in cells, for eample, in physiological, chemical, as well as biochemical activities (Todeschini et al., 2011; Marschner, 2012). Micronutrients such as Zn and Si have been found to provide several benefits under various crop conditions, including enhancing the N uptake and using efficiency and increasing production of crop biomass over the NPK levels (Angle et al., 2017). Zn NPs play a crucial role in various anatomical and physiological processes in plants (Agarwal et al., 2017). ZnO is essential for the activity of many enzymes, including dehydrogenases and superoxide dismutase (Narendhran et al., 2016). ZnO NPs are utilized as chemical absorbents, antibacterial agents, catalysts, and polymer additives due to their low toxicity, large specific surface area, high pore volume, long lifespan, and photodegradability (Abbasi Khalaki et al., 2021). Thus, zinc is vital for chlorophyll and biomass production, pollen function, RNA metabolism, and DNA formation (Pandey et al., 2006; Cakmak, 2008).

The used levels of ZnO NPs and SiO_2_ NPs did not exhibit any evidence of toxicity on the sage plant. Previous researches on other plant species including *Alyssum desertorum*, *Borago officinalis*, *Calendula officinalis*, and *Thymus vulgaris* (Yadegari, 2017), documented that Zn and Fe had improving impacts on the growth parameters of such plants. ZnO NPs increased chlorophyll pigments in *Borago officinalis* (Mohammadi et al., 2018). Likewise, on *Origanum majorana* El-Khateeb et al. (2020), on rosemary Hassanpouraghdam et al. (2020), and on *Mentha piperita* Lafmejani et al. (2021). They found that spraying of nano zinc singly or together with other elements enhanced the vegetative characteristics of such plants. They added that the effect of nano zinc was related to the level, kinds of plants, and utilization time (plant age). It has been reported that 6 g/L Zn NPs gave the maximum fresh and dry weights of sweet basil shoots in relation to 0, 2, and 4 mg/L Zn NPs or 2, 4, and 6 mg/L of either iron NPs or potassium NPs (Danaee and Abdossi, 2021). According to Khan et al. (2023), ZnO at 10 mg/L induced more biomas and chlorophyll content of *Echinops macrochaetus* than the untreated plants. Additionally, El-Mahrouk et al. (2024) found that 2g/L nano ZnO +20g/L date pollen extract, combined with half dose of the recommended NPK fertilizer was the most positive and effective treatment for the vegetative traits of sweet basil. Nano zinc at 10–800 mg/kg soil improved the quality of potato tubers (Zhang et al., 2024). The beneficial impact of nano SiO_2_ on plant metabolism leads to improved sage growth efficiency. Silicon nanoparticles possess physiological characteristics that enable them to affect a plant’s metabolic processes (Rastogi et al., 2019). SiO_2_ NPs are capable of transferring elements and DNA into plant tissues (Torney et al., 2007). Additionally, nano-silica enhances germination rates, radicle height, and plant dry weight (Azimi et al., 2014). SiO_2_ NPs also improve nutrient availability in maize (Suriyaprabha et al., 2012).

Additionally, silicon spraying affected the relative chlorophyll content of orchids (Mantovani et al., 2018). The development of *Arabidopsis thaliana* seedlings was stimulated with use of 10 mg/L SiO_2_ NPs and the chlorophyll level was increased to the maximum rate of 500 mg/L (Azhar et al., 2021). The spraying of silicon or nitric oxide, especially a combination of them, boosted leaf chlorophyll, which led to an improvment in photosynthesis, and in turn accounted for the weight of shoots of the sage plant under Cu-stress (Pirooz et al., 2021). Fenugreek plants treated with 200 kg/ha NPK (20:20:20) + NPK NPs at 2 g/L (20:20:20) + 2 g/L of nano elements (B, Mo, Cu, at 0.5%, Zn and Mg at 1.5%, and Fe at 8%) + nano seaweed extract at 2 g/L resulted in maximum values of the growth characteristics, when compared to untreated plants (Al-Saidi et al., 2022). Also, SiO_2_ NPs at 250, 500, and 1000 mg/L could prove the significant efficiency in seed germination and growth seedling length of *Calendula officinalis* and *Mellisa officinalis* (Bovand et al., 2023). Silicon NPs at 500 mg/kg soil improved root, shoot, length, as also the chlorophyll content of *Eruca sativa* (Mathur and Goswami, 2024). SiO_2_ NPs at 6, 12, and 18 g/L significantly increased the number and fresh and dry weights of shallot tubers (Rahmawati and Wulandari, 2024). Notably, NPs can have varying impacts on plant growth and development, depending on factors such as plant species and the characteristics of the nanoparticles, including their nature, composition, reactivity, and dosage (Khodakovskaya et al., 2012).

Enhancing impacts of DPE foliar sprays on sage growth and development were demonstrated in our results, which align with the previously mentioned constituents of DPE. In general, many authors have documented the improving impact of DPE on the development of plant species, attributing it to the presence of auxins and cytokinins in pollen (Alférez et al., 2000; Merwad et al., 2015). Hassan (2011) found that number, length, and weights (fresh and dry) of banana shoots were increased as a result of adding 200 mg/L of water pollen extract to a tissue culture medium. Similarly, Abou-Sreea and Yassen (2016) revealed that 20 g/L DPE had the greatest impact on improving the vegetative parameters of *Strelitzia reginae*, compared to the control with 5-15 g/L of DPE.

Based on the previous data, there are several factors, such as, climatic conditions and environmental factors, which can affect the EO% (Russo et al., 2013). The agricultural practices such as fertilization, controlling of weeds, insects, etc., as well as temperature and flowering stage development at harvesting time can positively regulate EO content (Hassiotis et al., 2014). Seemingly a balance had occured among macro- and micro-nutrients as a consequence of adding NPK, NPs of ZnO and SiO_2,_ or DPE (that contains several constituents). This balance may enhance various physiological, chemical, and biochemical processes or enzymatic activities in plant cells, including secondary metabolism, leading to increased EO synthesis. The concentrations of Ca^++^ and K^+^ strongly affect the EO yield (Yavari et al., 2010). According to Alhasan (2020), there is a non-linear relationship between the NPK rate and basil EO (%, v/w). Zinc plays a role in carbohydrate synthesis, protein metabolism, and auxin regulation, which in turn affects the EO content (Broadley et al., 2007). Furthermore, the positive effect of nano Zn on EO productivity, individually or in combination with another element, has been documented in studies on *Pimpinella anisum* (Pirzad and Barin, 2018), *Origanum majorana* (El-Khateeb et al., 2020), *Brassica nigra* (Zafar et al., 2020), and *Mentha piperita* (Lafmejani et al., 2021). Si stimulated secondary metabolism content of many species of plants (Ahanger et al., 2020; Santisree et al., 2020). Furthermore, Si is involved in EO synthesis, as demonstrated by numerous studies on basil (Farouk and Omar, 2020) and rose (Farahani et al., 2021) under drought stress and sage under Cu stress (Al-Saidi et al., 2022). Currently, there is very limited literature available on the use of DPE on medicinal and aromatic plants. However, it can be inferred that the improvement in sage EO synthesis may have been caused by the stimulative influences of various chemical and biochemical constituents of DPE on the different metabolic activities of plant cells, leading to enhanced essential oil synthesis.

It was noticed that higher percentages of monoterpene hydrocarbons, total compounds, and oxygenated hydrocarbons had resulted from usage of 75% NPK, combined with lower levels of DPE, SiO_2_ NPs, and ZnO NPs, respectively; even as a higher percentage of sesquiterpene hydrocarbons were observed in the plants EOthat received 50% NPKplus high levels of DPE and ZnO NPs. The synthesis of these compounds might be correlated with the concentrations of elements in the fertilization treatments used and the balance between the NPK level and DPE or concentrations of the NPs used. Although N is a necessary component of secondary metabolism, P has the necessary roles for the transport of carbohydrates and energy in leaf cells (Hawkesford et al., 2012). K has a role in a number of essential plant metabolic activities that enhance phloem transport, osmatic equilibrium, and photosynthesis (Hamzei et al., 2014). Additionally, NPs stimulate secondary metabolism such as terpenoid compounds (Mei et al., 2020) and promoted gene expression of secondary metabolism (Wang et al., 2021). DPE constituents have an impact on the metabolic processes, leading to elevated secondary metabolism (El-Mahrouk et al., 2024) in sweet basil. In the sage plant (Amer et al., 2019), in which NPK at 100%, 75%, and 50% alone or in combination with a biofertilizer (N-fexing bacteria and phosphate solubilizing bacteria) differently affect the total EO-identified compounds ranged from 9 to 22, comprising 83.6% to 99.9% of EO, including monterpenes (0% to13.4%), oxygenated monoterpens (83.4% to96.8%), sesquiterpenoids (0% to4.1%), oxygenated sesquiterpens (0% to0.9%), and oxygenated diterpens (0% to0.8%), and the highest α–thujone of 56.2% was observed with 100% NPK combined with biofertilizer, while 75% NPK with biofertilizer showed the highest percetntage of B-thujone (55.8%) and camphor (29.2%). Also, Sharma et al. (2019) mentioned that 49 aromatic compounds were observed in sage EO and the principal constituents were 1,8- cineole, camphor, α -thujone, α -humulene, rosmaric acid, and queretin. Basil EO components (percentage and amount) were impacted by nano-chelate fertilizers (K, F, and Zn) at 2–6 mg/L (Danaee and Abdossi, 2021).

Total phenolic compounds and antioxidant activity essentially have beneficial roles in plant protection. TPCs have significant roles in plants because of their ability to scavenge free radicals, which is attributed to their hydroxyl groups. Consequently, the presence of plant TPCs can be immediately linked to their AOA (Tosun et al., 2009). Moreover, it has been established that phenols possess antioxidant characteristics and are employed in the enzyme activity system and principle metabolic production (Jokar and Ronaghi, 2015). Additionally, studies on sunflower (Kiarostami et al., 2010), rosemary (Rady et al., 2011), lavender (Chrysargyris et al., 2018), and sweet basil (Ahmed et al., 2019) have demonstrated an association between TPCs and antioxidant activity that is good for plants.

According to Lingyun et al. (2016), the beneficial impacts on AOA may be attributed to important zinc factions in phenolic synthesis, maintaining membrane integrity, enhancing the scavenging molecule levels, protecting fundamental molecules, preserving groups of sulfhydryl, and preventing unnecessary interactions between iron and groups of other chemicals. Thus, zinc has an important function of maintening cell membranes under harmful conditions. Application of Zn or Zn NPs at various levels increased TPCs in various plant species like basil (Fallahi et al., 2016; Danaee and Abdossi, 2021), *Chrusanthemum balsamita* (Derakhshani et al., 2011), and rosemary (Hassanpouraghdam et al., 2020). Also, Zn improved the antioxidant system in wheat (Adrees et al., 2021). Similarly, ZnO NPs alone or in combination with Si NPs, positively influenced antioxidant enzyme activity in two *Brassica napus* species (El-Badri et al., 2021).

Furthermore, Nourozi et al. (2019) documented that SiO_2_ NPs can enhance TPC and flavonoid content, rosmarinic acid, and xantomicrol, and raise AOA in the hairy roots of *Dracocephalum kotschyi* Boiss via upregulated rosmarinic acid synthase and phenylalanine ammonia layse (PAL) expression genes. Over and above all this, the role of Si in improving TPC biosynthesis in cells of plants by enhancing the enzymatic activities conjunct in the pathway of phenylpropanoid, such as PAL, has been recognized (Ahanger et al., 2020). Moreover, treating *Salvia officinalis* plants with 1µM Si or 200 µM Si NPs has been shown to enhance TPCs and the DPPH scavenging activity (Al-Saidi et al., 2022). The application of 75% NPK + biofertilizer led to improvements in total phenol content and antioxidant activity compared to NPK at 50% and 100% (Amer et al., 2019). Additionally, K NPs have been found to have a positive effect on total phenol contents in basil (Danaee and Abdossi, 2021). Total phenols and antioxidant activity biosynthesis have been significantly increased in leaves of sweet basil, by applying different combinations of three-fourth or one-half of an NPK dose, with ZnO NPs, SiO_2_NPs, and date pollen extract, in comparison to a full dose of NPK (El-Mahrouk et al., 2024). Silicon oxide NPs at 100–1000 mg/kg soil raised the total protein, phenolic and flavonoid contents, and antioxidants in comparison to the control plants of *Eruca sativa* (Mathur and Goswami, 2024).

Our study focused on the chemical composition of sage leaves to evaluate the role of various applications on the percentages of nitrogen (N), phosphorus (P), potassium (K), zinc (Zn), silicon (Si), and total carbohydrates. The concentrations of these parameters in leaves under different treatments may depend on their levels and the balance between them in the applications. Zinc can be stored in plant leaves through ZnO nanoparticle (NP) vegetative applications, and these nanoparticles may act as an active zinc source in plant metabolism (Li et al., 2018, 2019). Zinc plays a crucial role in the synthesis of carbohydrates and proteins (Soliman et al., 2015). Additionally, Sadak and Bakry (2020) demonstrated that ZnO NPs at 20, 40, and 60 mg/L gradually and significantly increased total carbohydrates in *Linum usitatissimum* compared to the control. Similarly, Hassanpouraghdam et al. (2020) found that foliar spraying of Zn NPs at 3 mg/L increased rosemary Zn content, while the highest K content was observed in the control. Notably, foliar sprays of Zn and K nano-chelates at 2, 4, and 6 mg/L increased basil Zn and K contents, respectively, compared to the control (Danaee and Abdossi, 2021). A study on *Pimpinella anisum* by Pirzad and Barin (2018) found a significant increase in leaf Zn with 6 g/L of Fe+Zn foliar spraying, with maximum leaf N at 2 g/L Fe + 6 g/L Zn, and the highest leaf P and K contents at 4 g/L Zn. Nano Zn foliar spray at 50 and 100 mg/L increased total carbohydrates in marjoram more than in untreated plants (El-Khateeb et al., 2020). Furthermore, Zafar et al. (2020) reported a steady increase in Zn content in all parts of *Brassica nigra* after applying ZnO NPs at 200-600 mg/kg soil. Zinc content also increased in various tissues of potato grown in soil containing 10-800 mg ZnO NPs/kg soil (Zhang et al., 2024). Additionally, Si foliar spraying improved uptake and increased Si content in Phalaenopsis and *Dendrobium orchids* (Mantovani et al., 2018).

There is great interest in using DPE because it contains several compounds, including macro and micro-elements, which enhance the elements in plant tissues. This was supported by Abou-Sreea and Yassen (2016) showed that 10g/L DPE foliar application stimulated the uptake and concentrations of N, P, K and total carbohydrates in *Strelitzia reginae* relative to control. Amer et al. (2019) treated sage with a 75% NPK dose plus *Azotobacter chroococcum*, *Bacillus megaterium* var. phosphaticum, and *B. cereus* resulting in the maximum carbohydrate value compared to the control, and 50 and 100% NPK alone or together with biofertilizer. Thus, the utilization of macronutrients such as N, P, K, Mg, S, and Ca, in combination with NPs, allows for precise nutrient delivery to plants while reducing the bulk requirements and associated costs (Ditta and Arshad, 2016; Chhipa, 2017).

## 5 Conclusions

The current research suggests the positive benefits of spraying NPs of ZnO and SiO_2_ and natural extracts, such as DPE. These can be used as partial substitutes of traditional NPK fertilizers. Whereas, ZnO NPs, SiO_2_ NPs, and DPE at various levels, combined singly or together with 75%, 50%, and 25% NPK RD have proved to be effective in enhancing the growth traits (plant height, chlorophyl index, and aerial fresh and dry weights/plant), EO productivity (EO% and yield, and chemical composition of EO), biochemical composition (TPCs and AOA) and leaf chemical composition (N, P, K, Zn, Si, and total carbohydrates percentages)of the sage (*Salvia officinalis*) plant, in relation to 100% NPK RD, with some exceptions. The used treatments have varied impacts on the studied traits, with significant differences among themselves in most cases. Fifty-eight compounds which were distributed among the fertilization treatments were identified and ranged from 11 to 32. Also, the fertilization treatments had different effects on the EO composition (monoterpene hydrocarbons, sesquiterpene hydrocarbons, oxygenated hydrocarbons, and their constituents). Consequently, the application of nanoparticles of various elements and DPE could be considered, to reduce the excessive utilization of conventional fertilizers, with the aim of producing safe medicinal and aromatic products. A future study on sage will be regarding the effects of NPs of some nutrients and natural extracts on sage production, under different environmental conditions.

## Data Availability

The raw data supporting the conclusions of this article will be made available by the authors, without undue reservation.

## References

[B1] Abbasi Khalaki M. Moameri M. Asgari Lajayer B. Astatkie T. (2021). Influence of nano-priming on seed germination and plant growth of forage and medicinal plants. Plant Growth Regulat. 93, 13–28. doi: 10.1007/s10725-020-00670-9

[B2] Abou-Sreea A. I. B. Yassen A. A. (2016). Pollen extracts application as a natural growth substance on *Strelitzia reginae* Ait. plants. Int. J. PharmTechnol. Res. 9, 16–23.

[B3] Acharya P. Jayaprakasha G. K. Crosby K. M. Jifon J. L. Patil B. S. (2020). Nanoparticle-mediated seed priming improves germination, growth, yield, and quality of watermelons (*Citrullus lanatus*) at multi-locations in Texas. Sci. Rep. 10, 5037. doi: 10.1038/s41598-020-61696-7 32193449 PMC7081193

[B4] Adhikari T. Kundu S. Rao A. S. (2013). Impact of SiO_2 and_ Mo nano particles on seed germination of rice (*Oryza sativa* L.). Int. J. Agric. Food Sci. Technol. 4, 809–816.

[B5] Adrees M. Khan Z. S. Hafeez M. Rizwan M. Hussain K. Asrar M.. (2021). Foliar exposure of zinc oxide nanoparticles improved the growth of wheat (*Triticum aestivum* L.) and decreased cadmium concentration in grains under simultaneous Cd and water deficient stress. Ecotox. Environ. Saf. 208, 111627. doi: 10.1016/j.ecoenv.2020.111627 33396147

[B6] Agarwal H. Kumar S. V. Rajeshkumar S. (2017). A review on green synthesis of zinc oxide nanoparticles–An eco-friendly approach. Resource-Efficient Technol. 3, 406–413. doi: 10.1016/j.reffit.2017.03.002

[B7] Ahanger M. A. Bhat J. A. Siddiqui M. H. Rinklebe J. Ahmad P. (2020). Integration of silicon and secondary metabolites in plants: a significant association in stress tolerance. J. Exper. Bot. 71, 6758–6774. doi: 10.1093/jxb/eraa291 32585681

[B8] Ahmed A. F. Attia F. A. Liu Z. Li C. Wei J. Kang W. (2019). Antioxidant activity and total phenolic content of essential oils and extracts of sweet basil (*Ocimum basilicum* L.) plants. Food Sci. Hum. Wellness 8, 299–305. doi: 10.1016/j.fshw.2019.07.004

[B9] Alférez M. J. Campos M. S. Haro A. López-Aliaga I. Lisbona F. Barrionuevo M. (2000). Beneficial effect of pollen and/or propolis on the metabolism of iron, calcium, phosphorus, and magnesium in rats with nutritional ferropenic anemia. J. Agric. Food Chem. 48, 5715–5722. doi: 10.1021/jf000635h 11087544

[B10] Alhasan A. S. (2020). Effect of different NPK nano-fertilizer rates on agronomic traits, essential oil, and seed yield of basil (*Ocimum basilicum* l. cv Dolly) grown under field conditions. Plant Arch. 20, 2959–2962.

[B11] Al-Helal A. A. (1992). Electrophoretic analysis of three selected isoenzymes of date palm pollen grains. Bot. Bull. Acad. Sin. 33, 241–246.

[B12] Ali A. Ambreen S. Javed R. Tabassum S. Ul Haq I. Zia M. (2017). ZnO nanostructure fabrication in different solvents transforms physio-chemical, biological and photodegradable properties. Materi. Sci. Engin: C 74, 137–145. doi: 10.1016/j.msec.2017.01.004 28254278

[B13] Al-Saidi A.-R. Al-Mohammad M. H. Al-Juthery H. W. (2022). “Effect of spraying some nano-fertilizers and their combinations on the growth and yield of fenugreek (*Trigonella foenum-graecum* L.),” in Electrochemical society advancing solid state & electrochemical science & technology. Monterial Canada: ECS UNITED, 012041.

[B14] Amer H. M. Salem S. H. Salaheldin S. Hussein M. S. Abd El-Fatah S. I. (2019). The growth, chemical composition and evaluation of antimicrobial activity of *Salvia officinalis* oil under partial substitution of mineral NPK fertilizer by bio-fertilizer. Mid. East J. 8, 457–468.

[B15] Angle J. Singh U. Dimkpa C. Heliums D. Bindraban P. (2017). Role of fertilisers for climate-resilient agriculture Vol. 802 (London, UK: Int. Fertiliser Soci), 44.

[B16] Auld D. S. (2001). “Zinc coordination sphere in biochemical zinc sites,” in ed. Maret W. Zinc Biochemistry, Physiology, and Homeostasis. Dordrecht: Springer, 85–127. doi: 10.1007/978-94-017-3728-9_6 11831461

[B17] Azhar B. J. Noor A. Zulfiqar A. Zeenat A. Ahmad S. Chishti I.. (2021). Effect of ZnO, SiO_2_ and composite nanoparticles on *Arabidopsis thaliana* and involvement of ethylene and cytokinin signaling pathways. Pak. J. Bot. 53, 437–446. doi: 10.30848/PJB2021-2(40)

[B18] Azimi R. Borzelabad M. J. Feizi H. Azimi A. (2014). Interaction of SiO_2_ nanoparticles with seed prechilling on germination and early seedling growth of tall wheatgrass (*Agropyron elongatum* L.). Polish J. Chem. Technol. 16, 25–29. doi: 10.2478/pjct-2014-0045

[B19] Basuny A. M. Arafat S. M. Soliman H. M. (2013). Chemical analysis of olive and palm pollen: Antioxidant and antimicrobial activation properties. Wudpecker J. Food Technol. 1, 14–21.

[B20] Binsan W. Benjakul S. Visessanguan W. Roytrakul S. Tanaka M. Kishimura H. (2008). Antioxidative activity of Mungoong, an extract paste, from the cephalothorax of white shrimp (*Litopenaeus vannamei*). Food Chemist. 106, 185–193. doi: 10.1016/j.foodchem.2007.05.065

[B21] Black C. Evans D. Ensminger L. White J. Clark F. (1965). “Chap. 18, water capacity,” in Methods of soil analysis part 1. Ed. Klute A. (Amer. Soc. Agron, Madison, Wisconson, USA).

[B22] Bovand F. Chavoshi S. Ghorbanpour M. (2023). Titanium dioxide and silicon dioxide nanoparticles differentially affect germination and biochemical traits in marigold (*Calendula officinalis* L.) and lemon balm (*Melissa officinalis* L.). Nanotechnol.Environ. Engin. 8, 281–295. doi: 10.1201/9781003375104

[B23] Bremner J. M. Mulvaney C. (1983). “Nitrogen—total,” in Eds. Page A. L. Miller R. H. Keeny D. R. Methods of soil analysis: Part 2. Chemical and microbiological properties, vol. 9. (Madison, Wisconsin: American Society of Agronomy. Soil Sci. Soc. Ameri.), 595–624.

[B24] Broadley M. R. White Pj Hammond Jp Zelko I. Lux A. (2007). Zinc in plants. New Phytol. 173, 677–702. doi: 10.1111/j.1469-8137.2007.01996.x 17286818

[B25] Cakmak I. (2008). Enrichment of cereal grains with zinc: agronomic or genetic biofortification. Plant Soil 302, 1–17. doi: 10.1007/s11104-007-9466-3

[B26] Cele T. (2020). “Preparation of nanoparticles,” in Silver nanoparticles-health and safety, vol. 15. (London, unitedkingdom: IntechOpen).

[B27] Chemists A. (1990). Official methods of analysis. 15th ed Vol. I (Arlington, VA: AOAC), 489.

[B28] Chen J. Wei X. (2018). Controlled-release fertilizers as a means to reduce nitrogen leaching and runoff in container-grown plant production. Nitrogen Agric. Updates 33, 33–53. doi: 10.5772/68163

[B29] Chhipa H. (2017). Nanofertilizers and nanopesticides for agriculture. Environ. Chemist. Lett. 15, 15–22. doi: 10.1007/s10311-016-0600-4

[B30] Chrysargyris A. Michailidi E. Tzortzakis N. (2018). Physiological and biochemical responses of *Lavandula angustifolia* to salinity under mineral foliar application. Front. Plant Sci. 9, 489. doi: 10.3389/fpls.2018.00489 29731759 PMC5920160

[B31] Congreves K. Van Eerd L. (2015). Nitrogen cycling and management in intensive horticultural systems. Nutr. Cycling Agroecosyst. 102, 299–318. doi: 10.1007/s10705-015-9704-7

[B32] Cools N. De Vos B. (2011). Availability and evaluation of European forest soil monitoring data in the study on the effects of air pollution on forests. Forest-Biogeosci. Forest. 4, 205. doi: 10.3832/ifor0588-004

[B33] Cottenie A. Verloo M. Kiekens L. Velghe G. Camerlynck R. (1982). Chemical analysis of plants and soils. Lab. Agroch. State Univ. Gent Belgium 63, 80–284.

[B34] Danaee E. Abdossi V. (2021). Effect of foliar application of iron, potassium, and zinc nano-chelates on nutritional value and essential oil of Basil (*Ocimum basilicum* L.). Food Health 4, 13–20.

[B35] Derakhshani Z. Hassani A. Sadaghiani M. H. R. Hassanpouraghdam M. B. Khalifani B. H. Dalkani M. (2011). Effect of zinc application on growth and some biochemical characteristics of costmary (*Chrysanthemum balsamita* L.). Communica. Soil Sci. Plant Anal. 42, 2493–2503. doi: 10.1080/00103624.2011.609257

[B36] Ditta A. Arshad M. (2016). Applications and perspectives of using nanomaterials for sustainable plant nutrition. Nanotechnol. Rev. 5, 209–229. doi: 10.1515/ntrev-2015-0060

[B37] El-Badri A. M. Batool M. Aa Mohamed I. Wang Z. Khatab A. Sherif A.. (2021). Antioxidative and metabolic contribution to salinity stress responses in two rapeseed cultivars during the early seedling stage. Antioxidants 10, 1227–1248. doi: 10.3390/antiox10081227 34439475 PMC8389040

[B38] El-Khateeb M. El-Attar A. Abo-Bakr Z. (2020). Effect of nano-microelements on growth, yield and essential oil production of sweet marjoram (*Origanum majorana*) plants. Plant Arch. 20, 8315–8324.

[B39] El-Mahrouk E.-S. M. Atef E. Gabr M. K. Aly M. A. Głowacka A. Ahmed M. A. (2024). Application of ZnO NPs, SiO_2_ NPs and date pollen extract as partial substitutes to nitrogen, phosphorus, and potassium fertilizers for sweet basil production. Plants 13, 172. doi: 10.3390/plants13020172 38256725 PMC10819998

[B40] Elmsellem H. Ouadi Y. E. Mokhtari M. Bendaif H. Steli H. Aouniti A.. (2019). A natural antioxidant and an environmentally friendly inhibitor of mild steel corrosion: a commercial oil of basil (*Ocimum basilicum* L.). J. .Chem. Technol. Metallurgy 54, 742–749.

[B41] El-Ramady H. Abdalla N. Alshaal T. El-Henawy A. Elmahrouk M. Bayoumi Y.. (2018). Plant nano-nutrition: perspectives and challenges. Nanotech. Food Secur. Water Treat., 129–161.

[B42] Esetlili B.Ç. Öztürk B. Çobanoğlu Ö. Anaç D. (2016). Sweet basil (*Ocimum basilicum* L.) and potassium fertilization. J. Plant Nutrit. 39, 35–44. doi: 10.1080/01904167.2015.1088022

[B43] Evenhuis B. de Waard P. (1980). Principles and practices in plant analysis. Fao. Soil Bull. 39, 152–156.

[B44] Faizan M. Bhat J. A. Chen C. AlYemeni M. N. Wijaya L. Ahmad P.. (2021). Zinc oxide nanoparticles (ZnO-NPs) induce salt tolerance by improving the antioxidant system and photosynthetic machinery in tomato. Plant Physiol. Biochem. 161, 122–130. doi: 10.1016/j.plaphy.2021.02.002 33581620

[B45] Fallahi A. Hassani A. Sefidkon F. (2016). Effect of foliar application of different zinc sources on yield and phytochemical characteristics of basil (*Ocimum basilicum* L.). Iranian J. Medic. Arom. Plants 32, 743–757. doi: 10.22092/ijmapr.2016.107858

[B46] Farahani H. Sajedi N. A. Madani H. Changizi M. Naeini M. R. (2021). Effect of foliar-applied silicon on flower yield and essential oil composition of damask rose (*Rosa damascena* Miller) under water deficit stress. Silicon 13, 4463–4472. doi: 10.1007/s12633-020-00762-1

[B47] Farouk S. Omar M. (2020). Sweet basil growth, physiological and ultrastructural modification, and oxidative defense system under water deficit and silicon forms treatment. J. Plant Growth Regul. 39, 1307–1331. doi: 10.1007/s00344-020-10071-x

[B48] Feregrino-Perez A. A. Magaña-López E. Guzmán C. Esquivel K. (2018). A general overview of the benefits and possible negative effects of the nanotechnology in horticulture. Scientia Hortic. 238, 126–137. doi: 10.1016/j.scienta.2018.03.060

[B49] García-Gómez C. García S. Obrador A. F. González D. Babín M. Fernández M. D. (2018). Effects of aged ZnO NPs and soil type on Zn availability, accumulation and toxicity to pea and beet in a greenhouse experiment. Ecotoxicol. Environ. Saf. 160, 222230. doi: 10.1016/j.ecoenv.2018.05.019 29807295

[B50] Gee G. Bauder J. (1986). “Particle-size analysis 1,” in Methods of soil analysis: Part 1—Physical and Mineralogical Methods. (Springer New York, NY), vol. 5, 383–411.

[B51] Genc Y. Mcdonald G. K. Graham R. D. (2006). Contribution of different mechanisms to zinc efficiency in bread wheat during early vegetative stage. Plant Soil 281, 353–367. doi: 10.1007/s11104-005-4725-7

[B52] Hamzei J. Najjari S. Sadeghi F. Seyedi M. (2014). Effect of foliar application of nano-iron chelate and inoculation with mesorhizobium bacteria on root nodulation, growth and yield of chickpea under rainfed conditions. Iranian J. Pulses Res. 5, 9–18. doi: 10.22067/IJPR.V1393I2.46895

[B53] Hassan H. M. (2011). Chemical composition and nutritional value of palm pollen grains. Global J. Biotechnol. Biochem. 6, 1–7.

[B54] Hassanpouraghdam M. B. Mehrabani L. V. Tzortzakis N. (2020). Foliar application of nano-zinc and iron affects physiological attributes of *Rosmarinus officinalis* and quietens NaCl salinity depression. J. Soil Sci. Plant Nutrit. 20, 335–345. doi: 10.1007/s42729-019-00111-1

[B55] Hassiotis C. Ntana F. Lazari D. Poulios S. Vlachonasios K. (2014). Environmental and developmental factors affect essential oil production and quality of *Lavandula angustifolia* during flowering period. Industr. Crops Prod. 62, 359–366. doi: 10.1016/j.indcrop.2014.08.048

[B56] Hawkesford M. Horst W. Kichey T. Lambers H. Schjoerring J. Møller I. S.. (2012). “Functions of macronutrients,” in Marschner's mineral nutrition of higher plants (Elsevier), 135–189.

[B57] Hench L. L. West J. K. (1990). The sol-gel process. Chem. Rev. 90, 33–72. doi: 10.1021/cr00099a003

[B58] Herbert D. Phipps P. Strange R. (1971). Determination of total carbohydrates. Methods Microbiol. 5, 290–344.

[B59] Hogendorp B. K. Cloyd R. A. Swiader J. M. (2012). Determination of silicon concentration in some horticultural plants. HortSci. 47, 1593–1595. doi: 10.21273/HORTSCI.47.11.1593

[B60] Jackson M. L. (1973). Soil chemical analysis, (India) Pvt. Ltd. (New Delhi: Prentice Hall), 498.

[B61] Jokar L. Ronaghi A. (2015). Effect of foliar application of different Fe levels and sources on growth and concentration of some nutrients in sorghum. J. Soil Plant Interactions-Isfahan Univ. Technol. 6, 163–174. doi: 10.18869/acadpub.ejgcst.6.2.163

[B62] Jones D. L. Cross P. Withers P. J. Deluca T. H. Robinson D. A. Quilliam R. S.. (2013). Nutrient stripping: the global disparity between food security and soil nutrient stocks. J. Appl. Ecol. 50, 851–862. doi: 10.1111/jpe.2013.50.issue-4

[B63] Kabata-Pendias A. (2000). Trace elements in soils and plants (Boca Raton: CRC press).

[B64] Kah M. Kookana R. S. Gogos A. Bucheli T. D. (2018). A critical evaluation of nanopesticides and nanofertilizers against their conventional analogues. Nat. Nanotechnol. 13, 677–684. doi: 10.1038/s41565-018-0131-1 29736032

[B65] Khan S. Al-Qurainy F. Al-Hashimi A. Nadeem M. Tarroum M. Shaikhaldein H. O.. (2023). Effect of green synthesized ZnO-NPs on growth, antioxidant system response and bioactive compound accumulation in echinops macrochaetus, a potential medicinal plant, and assessment of genome size (2C DNA content). Plants 12, 1669. doi: 10.3390/plants12081669 37111892 PMC10141689

[B66] Khare R. Upmanyu N. Shukla T. Jain V. Jha M. (2020). Compendium of *Salvia officinalis*: an overview. Curr. Tradit. Medic. 6, 300–311. doi: 10.2174/2215083805666190723095043

[B67] Khator K. Shekhawat G. (2020). Nitric oxide mitigates salt-induced oxidative stress in *Brassica juncea* seedlings by regulating ROS metabolism and antioxidant defense system. Biotech. 10, 499. 10.1007/s13205-020-02493-x PMC7603439 33150125

[B68] Khodakovskaya M. V. De Silva K. Biris A. S. Dervishi E. Villagarcia H. (2012). Carbon nanotubes induce growth enhancement of tobacco cells. ACS Nano. 6, 2128–2135. doi: 10.1021/nn204643g 22360840

[B69] Kiarostami Mohseni R. Saboora A. (2010). Biochemical changes of Rosmarinus officinalis under salt stress. J. Stress Physiol. Biochem. 6, 114–122.

[B70] Lafmejani Z. Jafari A. A. Moradi P. Ladan Moghadam A. (2021). Application of chelate and nano-chelate zinc micronutrient on morpho-physiological traits and essential oil compounds of peppermint (*Mentha piperita* L.). J. Medic. Plants By-product 10, 21–28. doi: 10.22092/JMPB.2020.125992.1109

[B71] Li C. Wang P. Lombi E. Cheng M. Tang C. Howard D. L.. (2018). Absorption of foliar-applied Zn fertilizers by trichomes in soybean and tomato. J. Exper. Bot. 69, 2717–2729. doi: 10.1093/jxb/ery085 29514247 PMC5920297

[B72] Li C. Wang P. van der Ent A. Cheng M. Jiang H. Lund Read T.. (2019). Absorption of foliar-applied Zn in sunflower (*Helianthus annuus*): importance of the cuticle, stomata and trichomes. Ann. Bot. 123, 57–68. doi: 10.1093/aob/mcy135 30020418 PMC6344099

[B73] Lingyun Y. Jian W. Chenggang W. Shan L. Shidong Z. (2016). Effect of zinc enrichment on growth and nutritional quality in pea sprouts. J. Food Nutr. Res. 4, 100–107.

[B74] Liu R. Lal R. (2015). Potentials of engineered nanoparticles as fertilizers for increasing agronomic productions. Sci. Total Environ. 514, 131–139. doi: 10.1016/j.scitotenv.2015.01.104 25659311

[B75] Loneragan J. F. Webb M. J. (1993). “Interactions between zinc and other nutrients affecting the growth of plants,” in Zinc in Soils and Plants: Proceedings The Int. Symp. on ‘Zinc in Soils and Plants’ held at The University of Western Australia, , 27–28 September, 1993. 119–134, Springer.

[B76] Lu S. Feng C. Gao C. Wang X. Xu X. Bai X.. (2016). Multifunctional environmental smart fertilizer based on L-aspartic acid for sustained nutrient release. J. Agric. Food Chem. 64, 4965–4974. 27244106 10.1021/acs.jafc.6b01133

[B77] Ma C. White J. C. Zhao J. Zhao Q. Xing B. (2018). Uptake of engineered nanoparticles by food crops: characterization, mechanisms, and implications. Ann. Rev. Food Sci. Technol. 9, 129–153. doi: 10.1146/annurev-food-030117-012657 29580140

[B78] Mahran G. Abdul-Wahab S. Attia A. (1985). “Constituents of the Egyptian date palm pollen: Saponin and lipid constituents of pollen grains,” in The Proceding of 1985 First Int. Conf Zagazig Univ.

[B79] Maksimović M. Vidic D. Miloš M. Šolić M. E. Abadžić S. Siljak-Yakovlev S. (2007). Effect of the environmental conditions on essential oil profile in two Dinaric *Salvia species: S. brachyodon* Vandas and *S. officinalis* L. Biochem. Systematics Ecol. 35, 473–478. doi: 10.1016/j.bse.2007.02.005

[B80] Mantovani C. De Mello Prado R. Pivetta K. F. L. (2018). Silicon foliar application on nutrition and growth of Phalaenopsis and Dendrobium orchids. Scientia Hortic. 241, 83–92. doi: 10.1016/j.scienta.2018.06.088

[B81] Marschner H. (1995). Mineral Nutrient of Higher Plants. edition 2 (London: Harcourt Brace and Company Publishers). Academic Pres s Limited.

[B82] Marschner P. (2012). Marschner's mineral nutrition of higher plants. 3rd edn (London: Academic Press).

[B83] Martínez-Esplá A. Zapata P. J. Castillo S. Guillén F. Martínez-Romero D. Valero D.. (2014). Preharvest application of methyl jasmonate (MeJA) in two plum cultivars. 1. Improvement of fruit growth and quality attributes at harvest. Postharvest Biol. Technol. 98, 98–105. doi: 10.1016/j.postharvbio.2014.07.011

[B84] Mathur J. Goswami P. (2024). Positive impact of green synthesized silica nanoparticles in plant growth promotion and physiological responses of *Eruca sativa* Mill. J.Soil Sci. Plant Nutrit. 24, 1–13. doi: 10.1007/s42729-024-01725-w

[B85] Mei Y. Sun H. Du G. Wang X. Lyu D. (2020). Exogenous chlorogenic acid alleviates oxidative stress in apple leaves by enhancing antioxidant capacity. Scientia Hortic. 274, 109676. doi: 10.1016/j.scienta.2020.109676

[B86] Merwad M. Mostafa E. Saleh M. Mansour A. (2015). Yield and fruit quality of Hayany date palm as affected by different pollen grain sources. Inter. J. Chem. Tech. Res. 8, 544–549.

[B87] Mohammadi H. Hatami M. Feghezadeh K. Ghorbanpour M. (2018). Mitigating effect of nano-zerovalent iron, iron sulfate and EDTA against oxidative stress induced by chromium in *Helianthus annuus* L. Acta Physiologiae Plantarum 40, 1–15. doi: 10.1007/s11738-018-2647-2

[B88] Murphy J. Riley J. P. (1962). A modified single solution method for the determination of phosphate in natural waters. Analytica Chimica Acta 27, 31–36. doi: 10.1016/S0003-2670(00)88444-5

[B89] Naddaf M. Kafa H. Ghanem I. (2020). Extraction and characterization of nano-silica from olive stones. Silicon 12, 185–192. doi: 10.1007/s12633-019-00112-w

[B90] Nagai T. Inoue R. Inoue H. Suzuki N. (2002). Scavenging capacities of pollen extracts from cistus ladaniferus on autoxidation, superoxide radicals, hydroxyl radicals, and DPPH radicals. Nutr. Res. 22, 519–526. doi: 10.1016/S0271-5317(01)00400-6

[B91] Narendhran S. Rajiv P. Sivaraj R. (2016). Toxicity of ZnO nanoparticles on germinating *Sesamum indicum* (Co-1) and their antibacterial activity. Bull. Materials Sci. 39, 415–421. doi: 10.1007/s12034-016-1172-4

[B92] Nelson D. W. Sommers L. E. (1996). “Total carbon, organic carbon, and organic matter,” in Methods of soil analysis: Part 3. Chemical Methods. Soil Science Society of America and American Society of Agronomy, vol. 5, 961–1010.

[B93] Noreen S. Fatima Z. Ahmad S. Athar H.-U.-R. Ashraf M. (2018). Foliar application of micronutrients in mitigating abiotic stress in crop plants. Plant Nutri. Abiotic Stress Tolera., 95–117.

[B94] Nourozi E. Hosseini B. Maleki R. Mandoulakani B. A. (2019). Pharmaceutical important phenolic compounds overproduction and gene expression analysis in *Dracocephalum kotschyi* hairy roots elicited by SiO2 nanoparticles. Indust. Crops Prod. 133, 435–446. doi: 10.1016/j.indcrop.2019.03.053

[B95] Olsen S. Sommers L. (1982). Phosphorus. Methods of soil analysis: Part 2. Chem. Microbiol. Properties. Agron. Monograph, 421–422.

[B96] Page A. Miller R. Keeney D. (1982). “Methods of soil analysis Part 2-Chem. Microbiol. Properties,” in Agronomy, vol. 9. (SSSA, Madison, USA).

[B97] Pandey A. Dalal S. Dutta S. Dixit A. (2021). Structural characterization of polycrystalline thin films by X-ray diffraction techniques. J.ornal Mat. Sci. Mat. Electron. 32, 1341–1368. doi: 10.1007/s10854-020-04998-w

[B98] Pandey N. Pathak G. C. Sharma C. P. (2006). Zinc is critically required for pollen function and fertilisation in lentil. J. Trace Elements Medic. Biol. 20, 89–96. doi: 10.1016/j.jtemb.2005.09.006 16785048

[B99] Pirooz P. Amooaghaie R. Ahadi A. Sharififar F. (2021). Silicon-induced nitric oxide burst modulates systemic defensive responses of *Salvia officinalis* under copper toxicity. Plant Physiol. Biochem. 162, 752–761. doi: 10.1016/j.plaphy.2021.02.048 33799186

[B100] Pirzad A. Barin M. (2018). Iron and zinc interaction on leaf nutrients and the essential oil of *Pimpinella anisum* L. Irani. J. Plant Physiol. 8, 2507–2515. doi: 10.30495/ijpp.2018.543275

[B101] Pitsikas N. (2016). Constituents of saffron (*Crocus sativus* L.) as potential candidates for the treatment of anxiety disorders and schizophrenia. Molecules 21, 303. doi: 10.3390/molecules21030303 26950102 PMC6273654

[B102] Prakash B. Kedia A. Mishra P. K. Dubey N. (2015). Plant essential oils as food preservatives to control moulds, mycotoxin contamination and oxidative deterioration of agri-food commodities–Potentials and challenges. Food Control 47, 381–391. doi: 10.1016/j.foodcont.2014.07.023

[B103] Prasad R. Bhattacharyya A. Nguyen Q. D. (2017). Nanotechnology in sustainable agriculture: recent developments, challenges, and perspectives. Front. Microbiol. 8, 1014. doi: 10.3389/fmicb.2017.01014 28676790 PMC5476687

[B104] Prasad T. Sudhakar P. Sreenivasulu Y. Latha P. Munaswamy V. Reddy K. R.. (2012). Effect of nanoscale zinc oxide particles on the germination, growth and yield of peanut. J. Plant Nutr. 35, 905–927. doi: 10.1080/01904167.2012.663443

[B105] Rady M. Sadak M. S. El-Bassiouny H. El-Monem A. (2011). Alleviation the adverse effects of salinity stress in sunflower cultivars using nicotinamide and α-tocopherol. Austral. J. Basic Appl. Sci. 5, 342–355.

[B106] Rahmawati N. Wulandari N. (2024). “Foliar application of SiO_2_ nanoparticles to increase shallot production under water stress as an effort to mitigate climate change,” in IOP Conference Series: Earth and Environ. Sci. 012031, IOP Publishing.

[B107] Rai-Kalal P. Jajoo A. (2021). Priming with zinc oxide nanoparticles improve germination and photosynthetic performance in wheat. Plant Physiol. Biochem. 160, 341–351. doi: 10.1016/j.plaphy.2021.01.032 33548801

[B108] Raliya R. Saharan V. Dimkpa C. Biswas P. (2017). Nanofertilizer for precision and sustainable agriculture: current state and future perspectives. J. Agric. Food Chem. 66, 6487–6503. doi: 10.1021/acs.jafc.7b02178 28835103

[B109] Rastogi A. Zivcak M. Tripathi D. Yadav S. Kalaji H. Brestic M. (2019). Phytotoxic effect of silver nanoparticles in *Triticum aestivum*: Improper regulation of photosystem I activity as the reason for oxidative damage in the chloroplast. Photosynthetica 57, 209–216. doi: 10.32615/ps.2019.019

[B110] Rizwan M. Ali S. Ur Rehman M. Z. Adrees M. Arshad M. Qayyum M. F.. (2019). Alleviation of cadmium accumulation in maize (*Zea mays* L.) by foliar spray of zinc oxide nanoparticles and biochar to contaminated soil. Environ. Poll. 248, 358–367. doi: 10.1016/j.envpol.2019.02.031 30818115

[B111] Russo A. Formisano C. Rigano D. Senatore F. Delfine S. Cardile V.. (2013). Chemical composition and anticancer activity of essential oils of Mediterranean sage (*Salvia officinalis* L.) grown in different environmental conditions. Food Chem. Toxicol. 55, 42–47. doi: 10.1016/j.fct.2012.12.036 23291326

[B112] Sadak M. S. Bakry B. A. (2020). Zinc-oxide and nano ZnO oxide effects on growth, some biochemical aspects, yield quantity, and quality of flax (*Linum uitatissimum* L.) in absence and presence of compost under sandy soil. Bull. Nat. Res. Centre 44, 1–12. doi: 10.1186/s42269-019-0259-7

[B113] Santisree P. Sanivarapu H. Gundavarapu S. Sharma K. K. Bhatnagar-Mathur P. (2020). Nitric oxide as a signal in inducing secondary metabolites during plant stress. Co-evolution Secondary Metabolites, 593–621. doi: 10.1007/978-3-319-96397-6_61

[B114] Scimeca M. Bischetti S. Lamsira H. K. Bonfiglio R. Bonanno E. (2018). Energy Dispersive X-ray (EDX) microanalysis: A powerful tool in biomedical research and diagnosis. Europ. J. Histochem: EJH 62, 62–72. doi: 10.4081/ejh.2018.2841 PMC5907194 29569878

[B115] Sharma A. Shahzad B. Rehman A. Bhardwaj R. Landi M. Zheng B. (2019). Response of phenylpropanoid pathway and the role of polyphenols in plants under abiotic stress. Molecules 24, 2413–2452. doi: 10.3390/molecules24132452 31277395 PMC6651195

[B116] Singleton V. L. Orthofer R. Lamuela-Raventós R. M. (1999). “[14] Analysis of total phenols and other oxidation substrates and antioxidants by means of folin-ciocalteu reagent,” in Methods in Enzymology (Elsevier), 152–178.

[B117] Snedecor G. W. Cochran W. G. (1989). “Statistical methods,” in The Journal of Agricultural Science, 8thEdn, vol. 115. (Iowa State Univ. Press Iowa, Ames), 153–153.

[B118] Solanki P. Bhargava A. Chhipa H. Jain N. Panwar J. (2015). Nano-fertilizers and their smart delivery system. Nanotechnol. Food Agric., 81–101.

[B119] Soliman A. S. El-Feky S. A. Darwish E. (2015). Alleviation of salt stress on *Moringa peregrina* using foliar application of nanofertilizers. J. Hortic. Forest. 7, 36–47. doi: 10.5897/JHF2014.0379

[B120] Sommer M. Kaczorek D. Kuzyakov Y. Breuer J. (2006). Silicon pools and fluxes in soils and landscapes—a review. J. Plant Nutr. Soil Sci. 169, 310–329. doi: 10.1002/jpln.200521981

[B121] Subramanian K. S. Manikandan A. Thirunavukkarasu M. Rahale C. S. (2015). Nano-fertilizers for balanced crop nutrition. Nanotechnol Food Agric., 69–80.

[B122] Suriyaprabha R. Karunakaran G. Yuvakkumar R. Rajendran V. Kannan N. (2012). Silica nanoparticles for increased silica availability in maize (*Zea mays.* L) seeds under hydroponic conditions. Curr. Nanosci. 8, 902–908. doi: 10.2174/157341312803989033

[B123] Swarnapriya R. Vaibhao Ramesh G. (2020). Role of nanomaterials in vegetable crops. E. Scientia Lett. 5, 35–42.

[B124] Takkar P. W. C. D. (1993). “The distribution and correlation of zinc deficiency,” in zinc in soils and plants, vol. 1993 . Ed. Robson A. D. (Kulmer Academic Publishers), 151–165. Proceedings The Int. Symp. on ‘Zinc in Soils and Plants, held at the University of Western Australia, 27-28 Septemper.

[B125] Todeschini V. Lingua G. D’agostino G. Carniato F. Roccotiello E. Berta G. (2011). Effects of high zinc concentration on poplar leaves: a morphological and biochemical study. Environ. Experi. Bot. 71, 50–56. doi: 10.1016/j.envexpbot.2010.10.018

[B126] Torney F. Trewyn B. G. Lin V. S.-Y. Wang K. (2007). Mesoporous silica nanoparticles deliver DNA and chemicals into plants. Nat. Nanotechnol. 2, 295–300. doi: 10.1038/nnano.2007.108 18654287

[B127] Tosun M. Ercisli S. Sengul M. Ozer H. Polat T. Ozturk E. (2009). Antioxidant properties and total phenolic content of eight *Salvia species* from Turkey. Biolog. Res. 42, 175–181. doi: 10.4067/S0716-97602009000200005 19746262

[B128] Vanlauwe B. Bationo A. Chianu J. Giller K. E. Merckx R. Mokwunye U.. (2010). Integrated soil fertility management: operational definition and consequences for implementation and dissemination. Outlook Agric. 39, 17–24. doi: 10.5367/000000010791169998

[B129] Wang Q. Su H. Yue N. Li M. Li C. Wang J.. (2021). Dissipation and risk assessment of forchlorfenuron and its major metabolites in oriental melon under greenhouse cultivation. Ecotox. Environ. Saf. 225, 112700. doi: 10.1016/j.ecoenv.2021.112700 34500385

[B130] Yadava U. L. (1986). A raped and non-destructive method to determined chlorophyll in intact leaves. Hortsci. 21, 1449–1450. doi: 10.21273/HORTSCI.21.6.1449

[B131] Yadegari M. (2017). Irrigation periods and Fe, Zn foliar application on agronomic characters of *Borago officinalis, Calendula officinalis, Thymus vulgaris* and *Alyssum desertorum* . Commun. Soil Sci. Plant Anal. 48, 307–315. doi: 10.1080/00103624.2016.1269796

[B132] Yavari A. Nazeri V. Sefidkon F. Hassani M. E. (2010). Influence of some environmental factors on the essential oil variability of *Thymus migricus* . Natural Product Commun. 5, 1934578X1000500629. doi: 10.1177/1934578X1000500629 20614832

[B133] Zafar H. Aziz T. Khan B. Mannan A. Rehman R. U. Zia M. (2020). CuO and ZnO nanoparticle application in synthetic soil modulates morphology, nutritional contents, and metal analysis of *Brassica nigra* . ACS Omega 5, 13566–13577. doi: 10.1021/acsomega.0c00030 32566821 PMC7301370

[B134] Zhang H. Zhao X. Bai J. Tang M. Du W. Lv Z.. (2024). Effect of ZnO nanoparticle application on crop safety and soil environment: a case study of potato planting. Environ. Sci. 11, 351–362. doi: 10.1039/D3EN00680H

